# Amyotrophic lateral sclerosis (ALS) and Alzheimer’s disease (AD) are characterised by differential activation of ER stress pathways: focus on UPR target genes

**DOI:** 10.1007/s12192-018-0897-y

**Published:** 2018-05-04

**Authors:** L. Montibeller, J. de Belleroche

**Affiliations:** Neurogenetics Group, Division of Brain Sciences, Faculty of Medicine, Imperial College London, London, UK

**Keywords:** UPR, ALS, AD, ERAD, Folding, ER stress

## Abstract

The endoplasmic reticulum (ER) plays an important role in maintenance of proteostasis through the unfolded protein response (UPR), which is strongly activated in most neurodegenerative disorders. UPR signalling pathways mediated by IRE1α and ATF6 play a crucial role in the maintenance of ER homeostasis through the transactivation of an array of transcription factors. When activated, these transcription factors induce the expression of genes involved in protein folding and degradation with pro-survival effects. However, the specific contribution of these transcription factors to different neurodegenerative diseases remains poorly defined. Here, we characterised 44 target genes strongly influenced by XBP1 and ATF6 and quantified the expression of a subset of genes in the human post-mortem spinal cord from amyotrophic lateral sclerosis (ALS) cases and in the frontal and temporal cortex from frontotemporal lobar degeneration (FTLD) and Alzheimer’s disease (AD) cases and controls. We found that IRE1α-XBP1 and ATF6 pathways were strongly activated both in ALS and AD. In ALS, XBP1 and ATF6 activation was confirmed by a substantial increase in the expression of both known and novel target genes involved particularly in co-chaperone activity and ER-associated degradation (ERAD) such as *DNAJB9*, *SEL1L* and *OS9*. In AD cases, a distinct pattern emerged, where targets involved in protein folding were more prominent, such as *CANX*, *PDIA3* and *PDIA6*. These results reveal that both overlapping and disease-specific patterns of IRE1α-XBP1 and ATF6 target genes are activated in AD and ALS, which may be relevant to the development of new therapeutic strategies.

**Electronic supplementary material:**

The online version of this article (10.1007/s12192-018-0897-y) contains supplementary material, which is available to authorized users.

**Graphical abstract:**
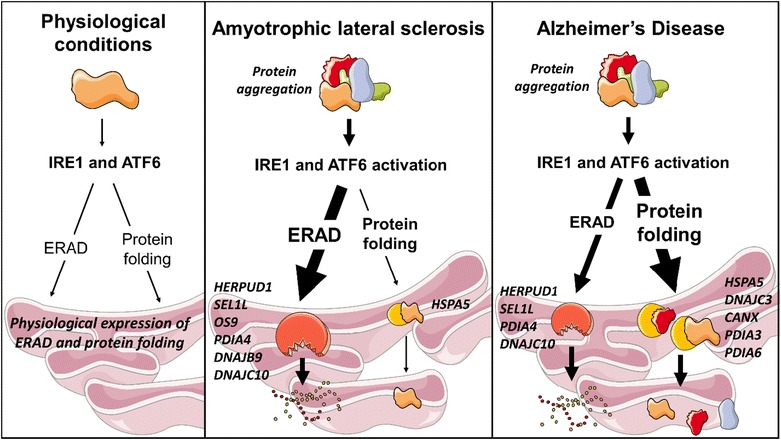
The endoplasmic reticulum (ER) plays an important role in maintenance of proteostasis through the unfolded protein response (UPR). Two major UPR signalling pathways are mediated by IRE1α and ATF6. Here, we demonstrate that these pathways activate differential gene sets in human post-mortem tissues derived from amyotrophic lateral sclerosis (ALS) compared to Alzheimer’s disease (AD) cases. Our results identify IRE1α and ATF6 specific targets that can have major implications in the development of new therapeutic strategies and potential biomarkers.

## Introduction

The majority of neurodegenerative diseases such as Alzheimer’s disease (AD), amyotrophic lateral sclerosis (ALS) and frontotemporal lobar degeneration (FTLD) are characterised by dysfunction in protein homeostasis (proteostasis) (Hetz and Saxena 2017). A major role in proteostasis is played by the endoplasmic reticulum (ER), which is responsible for the correct folding, modification and quality control of approximately one third of all secretory and membrane proteins (Hetz and Saxena 2017). When the protein folding demand exceeds the capacity of the cell to restore homeostasis, misfolded proteins accumulate in the ER leading to a condition known as ER stress. Markers of ER stress are evident in many neurodegenerative diseases indicating the involvement of common homeostatic mechanisms such as the unfolded protein response (UPR) (Kampinga and Bergink 2016).

The UPR is a system composed of three intimately connected signalling pathways, which are mediated by three ER membrane-resident sensors: the protein kinase RNA (PKR)-like ER kinase (PERK), the transmembrane basic leucine zipper activating transcription factor-6 (ATF6) and the evolutionary conserved kinase/endoribonuclease inositol-requiring protein-1 (IRE1α). Within the UPR, IRE1α and ATF6 pathways play a pro-survival role mediated by the induction of ER chaperones and proteins involved in the removal of misfolded proteins (Pincus et al. 2010; Shoulders et al. 2013). The regulation of these homeostatic genes is mediated by a set of related basic leucine zipper (bZIP) transcription factors which are activated by each UPR branch through unique signal transduction mechanisms (Travers et al. 2000). IRE1α is activated by autophosphorylation leading to the processing of X box-binding protein-1 mRNA, to yield (XBP1s), which is a potent transcription factor (TF) (Acosta-Alvear et al. 2007; Shoulders et al. 2013). In addition to XBP1, activated IRE1α is able to mediate and process the cleavage of cytoprotective mRNAs through a process called IRE1α-dependent decay (RIDD) (Maurel et al. 2014; Tam et al. 2014; Hetz and Saxena 2017). The ATF6 full-length protein, instead, is activated after translocation to the Golgi apparatus where it is processed and released as a soluble cytoplasmic fragment that is able to enter into the nucleus and activate the transcription of a specific subset of genes (Ye et al. 2000). There are several lines of evidence showing that IRE1α-XBP1 and ATF6 pathways are intimately inter-connected. The expression of XBP1 is partially regulated by ATF6 (Yoshida et al. 2001; Dunham et al. 2012). Moreover, IRE1α-XBP1 and ATF6 pathways regulate the transcriptional induction of a similar set of genes, which are characterised by the presence of specific *cis*-acting elements in their promoter regions (Acosta-Alvear et al. 2007; Yamamoto et al. 2007; Pincus et al. 2010; Shoulders et al. 2013). These *cis*-acting elements are recognised by XBP1 and can be divided into four main binding sites: ERSE (ER stress response elements, the consensus sequence of which is CCAAT-N9-CCACG) (Yoshida et al. 2001), ERSE-II (ER stress response element II, ATTGG-N1-CCACG), ERSE-26 (CCAAT-N26-CCACG) and UPRE (unfolded protein response element) (Yamamoto et al. 2004; Acosta-Alvear et al. 2007; Misiewicz et al. 2013). A highly conserved core region (ACGT core) which has been found as a single entity and within the UPRE sequence is also recognised by XBP1s (Kanemoto et al. 2005; Acosta-Alvear et al. 2007). Notably, several studies have demonstrated that ERSE and ERSE-II binding sites are also recognised by ATF6 (Yamamoto 2004), which may be able to hetero-dimerise with XBP1 activating different sets of genes such as *DNAJC3* and *HYOU1* (Newman 2003; Yamamoto et al. 2007; Shoulders et al. 2013). Thus, due to this close interaction, the specific contribution of IRE1α-XBP1 and ATF6 pathways in normal and pathological condition is still not well understood.

Recent studies have demonstrated an involvement of the IRE1α-XBP1 and ATF6 pathways in human neurodegenerative diseases. Phosphorylated IRE1α (p-IRE1α) and XBP1s have been found to be increased in different human brain regions of patients affected by AD (Hoozemans et al. 2009; Lee et al. 2010). The activation of the IRE1α-XBP1 pathway correlates with the presence of abnormally phosphorylated tau in AD neurons (Hoozemans et al. 2009). Furthermore, the molecular activation of this pathway in animal models of AD is involved in the prevention of Aβ toxicity through the regulation of specific gene targets such as *HRD1* (Casas-Tinto et al. 2011; Endres and Reinhardt 2013; Song et al. 2015). The IRE1α-XBP1 pathway has also been found to be activated in the human spinal cord of sporadic cases and in mouse models of ALS (Atkin et al. 2008). This activation was associated with elevated levels of ER chaperones such as PDIA1 and ERp57 (Ilieva et al. 2007; Hetz et al. 2009). Interestingly, the activation of this pathway represents one of the earliest pathological events in motor neurons, which occurs before the onset of symptoms in mouse models of ALS (Atkin et al. 2008). In addition, an increase in *XBP1* splicing and its translocation to the nucleus have been demonstrated to occur in motor neuronal cell lines expressing ALS-associated mutations (Prell et al. 2012). This evidence suggests that the IRE1α-XBP1 pathway is activated in AD and ALS by increasing the expression of a set of genes that remove aberrant proteins and restore protein homeostasis (Casas-Tinto et al. 2011). In contrast, the involvement of the ATF6 pathway in neurodegenerative disorders is poorly described and remains largely unknown (Xiang et al. 2017). In AD, a PS1 mutation inhibits the activation of ATF6 whereas protein levels of activated ATF6 increased in the spinal cord of sporadic ALS (Katayama et al. 2001; Atkin et al. 2008). In addition, both IRE1α-XBP1 and ATF6 transcriptional activity were found to be significantly attenuated in cell line models carrying an ALS-linked mutation in VAPB (Chen et al. 2010; Nardo et al. 2011). Thus, although this evidence supports the involvement of IRE1α-XBP1 and ATF6 pathways in AD and ALS, their specific role in these diseases remains poorly defined.

Here, we compared the IRE1α-XBP1 and ATF6 pathways in human post-mortem tissues of CNS in different neurodegenerative disorders. We found a differential activation of IRE1α-XBP1 and ATF6 target gene sets in ALS compared to AD cases including genes that had never been described before in these disorders. This study extends our understanding of the cellular mechanisms of ER stress involved in neurodegenerative disorders highlighting similarities and differences between IRE1α-XBP1 and ATF6 in AD and ALS, which can be used to identify therapeutic targets and to develop disease-specific targets/biomarkers.

## Materials and methods

Reagents, oligonucleotide primers and general methods for bioinformatic analyses are described in SI Materials and Methods.

### Subjects

Spinal cord samples were available for 49 samples: 32 sporadic ALS (SALS) cases with a median age at death of 68 years (range 24–85 years) and median post-mortem delay of 14 h (range 5–25 h) and 17 control cases with a median age at death of 66 years (range 20–91 years) and median post-mortem delay of 11 h (range 3–19 h). Frozen dorsolateral prefrontal cortex (PFC) and temporal cortex (TC) tissues were obtained from 79 subjects: 20 were healthy controls with only ageing-related changes and 20 were classified as Alzheimer’s disease (AD) cases and 39 as frontotemporal lobar degeneration cases. Of the 39 FTLD cases, 19 were familial FTLD cases with a *C9orf72* hexanucleotide repeat expansion (FTLD C9+ve), while 20 did not have a *C9orf72* hexanucleotide repeat expansion (FTLD C9-ve). Gender, age at death and post-mortem delay (PMD) of subjects are listed in the additional file: Table S1. More details can be found in SI Materials and Methods.

### Human tissue sample preparation

This study was approved by the Riverside Research Ethics Committee and was carried out according to their guidelines. For the spinal cord cases and controls, frozen lumbar tissue sampled at levels L3 to L5 was used. Characteristics of this tissue have been described in detail from the point of view of motor neuron counts, Nissl staining, immunohistochemistry of neuronal markers (ChAT, VAPB and DAO) and p62 as a marker of ubiquitinated protein inclusions typical of ALS (Paul et al. 2014). In addition, the glial marker, GFAP, is significantly upregulated in these ALS cases, indicative of astrocytic proliferation known to occur in ALS (Anagnostou et al. 2010). Frozen dorsolateral PFC and TC tissues were obtained from the MRC London Neurodegenerative Diseases Brain Bank, a member of the Brains for Dementia Research Network. All tissue was obtained from voluntary donors in compliance with the Mental Capacity Act (2005), and the Brain Bank has been approved by the National Research Ethics Service. All clinical diagnoses were confirmed neuropathologically at post-mortem. Dissected brain tissue was snap frozen, then stored at − 80 °C until further use. More details can be found in SI Materials and Methods.

### Selection of gene targets

We considered XBP1 as the transcription factor for the IRE1α pathway (Yoshida et al. 2001), ATF6 for the ATF6 pathway (Haze et al. 1999) and ATF4/NFE2L2 for the PERK pathway (Cullinan et al. 2003; Fels and Koumenis 2006). The gene names were extracted from three different databases (JASPAR, Reactome and TRUSST) selecting *Homo sapiens* genome 19 (hg19) as the background model. More details can be found in SI Materials and methods.

### *Cis*-acting element analysis

For each RefSeq sequence of XBP1 target gene, we investigated the presence of ER stress response elements (if a gene showed multiple RefSeq sequences, the prevalent transcript was considered). Within those sequences 20 kb upstream and 5 kb downstream, we looked for exact matches or one base mismatch to the ER stress response element (ERSE) (CCAAT-N9-CCACG), ERSE-II (ATTGG-N-CCACG), ERSE-26 (CCAAT-N26-CCACG) and the unfolded protein response element (UPRE) (TGACGTGG/A) sequences. More details can be found in SI Materials and Methods.

### mRNA extraction and RNA quality assessment

mRNA was extracted from frozen tissue samples and stored at − 80 °C. RNA was extracted from frontal and temporal cortex and spinal cord samples following the Direct-zol RNA mini prep (Zymo Research) protocol. RNA purity and integrity for all tissue samples was assessed by using multiple well-established methods. For all spinal cord samples (ALS cases and controls), we measured tissue pH, as brain pH is decreased by prolonged death and hypoxia but remains stable post-mortem and varies little across regions. In brief, pH was measured in frozen cortical tissue samples from all cases and controls. Median values of pH for ALS cases and controls were 6.3 and 6.4, respectively (Anagnostou et al. 2010). Assessment of mRNA purity/quality in the total RNA sample was based on measurement of A260/280 absorbance ratios in all mRNA extractions and were typically in the range of 1.83 to 1.94 (mean = 1.89) for spinal cord tissue and 1.77–2.05 (mean = 1.94) for frontal and temporal cortex tissues, indicating a high level of purity. Assessment of RNA integrity was based on the integrity of ribosomal RNAs (rRNAs). The Agilent 2100 Bioanalyser in conjunction with the RNA 6000 LabChip® was used for assessment of RNA integrity of total RNA extracts from the spinal cord. Total RNAs with 28S:18S rRNA ratios > 1.0 were considered suitable for qPCR analysis and the remainder were rejected from further study. Further information about RNA integrity was obtained from electrophoresis of all qPCR amplification products and analysis of thermal dissociation profiles to ensure that single uncontaminated products were obtained (Fig. S5-6). Procedures for cDNA synthesis and mRNA quantification by qPCR are described in SI Materials and Methods.

### Quantitative real-time PCR

qPCR was performed using the Power Up™ SYBr™ Green Master Mix with 6 μM of primers. Stratagene® MX3000p qPCR system Primer sequences and temperatures utilised for real-time PCR analysis are listed in the additional file: Table S2. There was no significant correlation between mRNA levels and age at death and post-mortem delay in the spinal cord or in the frontal and temporal cortex. Similarly, no significant findings were obtained when each group was analysed separately, except for one gene, calnexin, which showed a significant decline in SALS with age. More details can be found in SI Materials and Methods.

### Western blot analysis

Sections from frozen tissue blocks were cut (15 μm) on a cryostat (Bright Instruments), homogenised and lysed with lysis buffer (20 nM Tria-HCl, 137 mM NaCl, 10% glycerol, 1% NP40, 2 nM EDTA). Proteins were separated with 10% SDS-PAGE, and blotting was carried out using conditions specified for the antibodies. Antibodies used were anti-HERPUD1 (Cell Signalling Technology), anti-PDIA6 (Abcam), anti-HSPA5/GRP78 (Proteintech), anti-DNAJC10 (Proteintech) and anti-β actin (Proteintech). More details can be found in SI Materials and Methods.

### Statistical analysis

Statistical analyses were performed using GraphPad Prism 5 software (GraphPad, La Jolla, CA, USA). Prism and R were used to draw graphs. For bioinformatic analyses, modified Fisher’s exact *P* value (EASE) was used to determine the gene-enrichment analysis and Benjamini-Hochberg multiple test correction to calculate the false discovery rate (FDR). Only the corrected values with *P* < 0.01 were analysed. For the gene expression analyses, the D’Agostino-Pearson test was used to test the normality of the data distribution. Results were expressed as mean ± SEM unless otherwise indicated. All statistical analyses and calculation of *P* values were performed using either two-tailed Student’s *t* test or two-way ANOVA, followed by Tukey’s multiple comparison tests for multiple group comparisons. Non-normally distributed data were analysed by the corresponding nonparametric tests (Mann-Whitney *U* test and Kruskal-Wallis followed by Dunn’s post-test, respectively). All data were checked for the presence of outliers by performing the GraphPad ROUT test. Sample size was described in the legend of each figure. Where qPCR analysis was carried out in two batches, the means of each control group were compared and, if different, a normalisation factor was applied. A *P* value of less than 0.05 was considered significant.

## Results

### Systematic analysis of the unfolded protein response target genes and ER stress response elements of XBP1 and ATF6 in human CNS

**Fig. 1:**
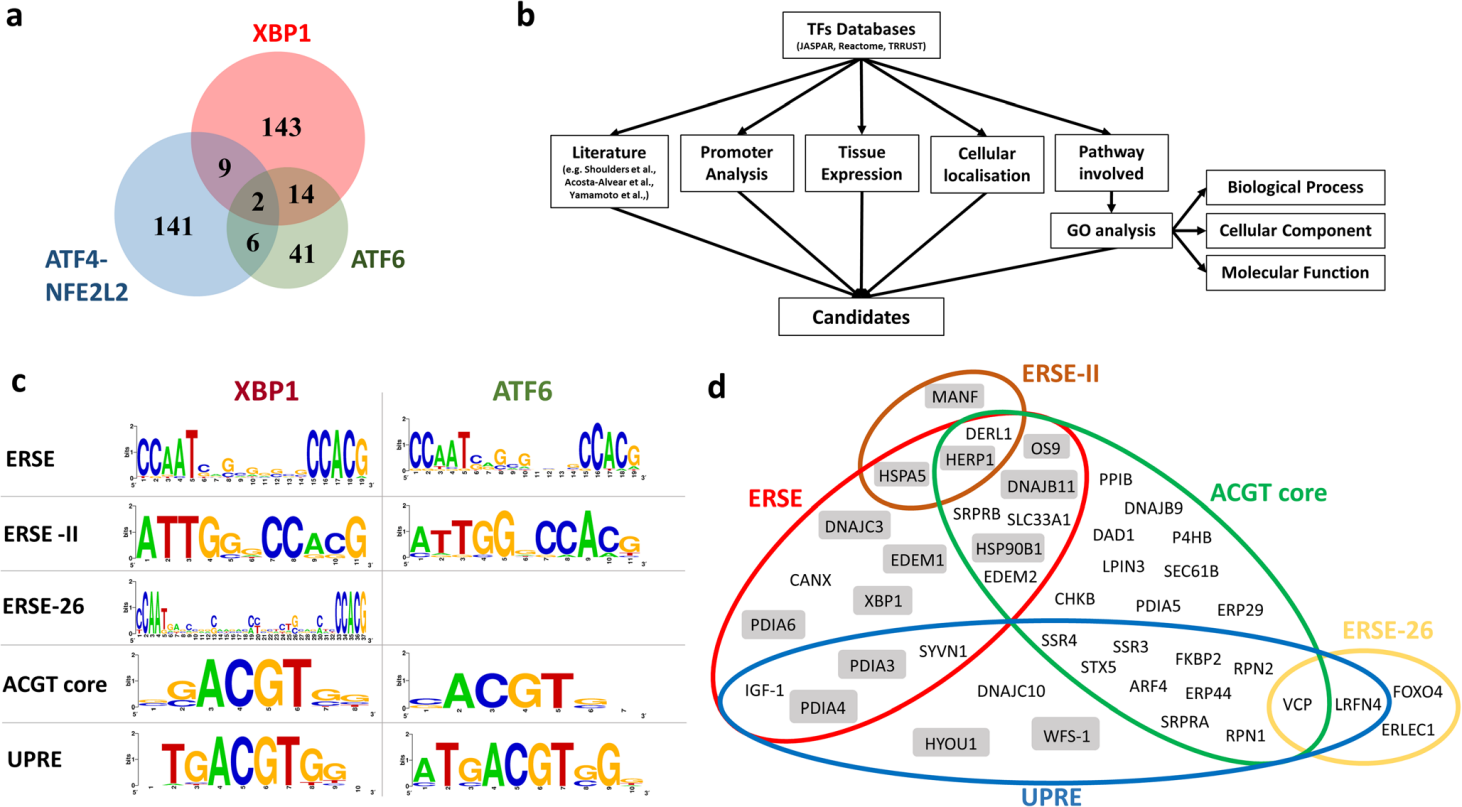
Systematic analysis of target genes of the UPR transcription factors XBP1, ATF6 and ATF4-NRF2. **a** Venn diagram showing the exclusive and overlapping target genes regulated by UPR TFs. The common genes between XBP1 and ATF4-NFE2L2 are 9, between XBP1 and ATF6 are 14, between ATF6 and ATF4-NFE2L2 are 6. Two genes were shared between all UPR TF. **b** Workflow for the selection of XBP1 and ATF6 target genes. **c** The 5 ER response elements identified in the promoter region of 44 XBP1 and ATF6 target genes were aligned using WebLogo (http://weblogo.berkeley.edu/). Each logo comprises stacks of symbols, one stack for each position in the sequence. The overall height of the stack indicates the sequence conservation at that position; the height of symbols within the stack indicates the relative frequency of each amino or nucleic acid at that position. A colour code is used for each base (blue, cytosine; red, thymidine; yellow, guanosine; green, adenosine). **d** The 44 XBP1 target genes listed in Table S4 were characterised and gathered based on the ER response elements present in their promoter. The genes inside the grey boxes were found to be regulated by both XBP1 and ATF6 according to the literature

The activation of the IRE1α-XBP1 and ATF6 pathways is triggered in ALS as well as other neurodegenerative diseases (Hetz and Saxena 2017). To characterise these specific responses in detail, we extrapolated the target genes of the main UPR arms (XBP1, ATF6 and PERK) from three different databases (JASPAR, Reactome and TRRUST). We considered XBP1 as the transcription factor for the IRE1α pathway (Yoshida et al. 2001), ATF6 for the ATF6 pathway (Haze et al. 1999) and ATF4/NFE2L2 for the PERK pathway (Cullinan et al. 2003; Fels and Koumenis 2006). We obtained 158 genes potentially regulated by ATF4-NFE2L2, 63 by ATF6 and 168 by XBP1s (additional file: Fig. S1A, Table S3). The target gene lists were merged in order to identify the common and the specific candidates that are regulated exclusively by each UPR pathway (Fig. 1a). To select the candidates that are regulated predominantly by IRE1α-XBP1 and ATF6, several parameters were used (Fig. 1b). Firstly, we refined the list of candidates considering the already well-established genes regulated by XBP1 (Acosta-Alvear et al. 2007; Sriburi et al. 2007; Dombroski et al. 2010; Shoulders et al. 2013) and ATF6 (Arai et al. 2006; Yamamoto et al. 2007; Adachi et al. 2008; Shoulders et al. 2013; Howarth et al. 2014) (additional file: Table S4). Secondly, we combined this information with the intra-cellular localisation and tissue expression of the candidates prioritising the genes that encode for proteins with ER localisation and neuronal expression (additional file: Table S4). Thirdly, we selected the candidate genes that are mainly involved in the protein homeostasis process, which were extrapolated from gene ontology (GO) enrichment analysis performed on the genes regulated by the UPR TFs (additional file: Fig. S1B-D, Table S5). Finally, we investigated the presence of ER stress/unfolded protein response elements (ERSE, ERSE-II, UPRE and ERSE-26) in promoter regions of the XBP1 and ATF6 target genes (Fig. 1c, d). Following these criteria, we selected 44 genes and we highlighted those that were regulated eitherby XBP1 or ATF6 or by both transcription factors (Fig. 1d, additional file: Table S4). Successively, a subset of 18 target genes was selected as a reference to understand the unique and the cooperative roles of XBP1 and ATF6. We included also a gene (*SIL1*) which is not regulated by neither ATF6 nor XBP1 although it is involved in protein folding processes. Finally, the expression of the these 19 genes was investigated in human brain frozen tissues derived by ALS, FTLD and AD cases relative to control cases (Table 1).

**Table 1:** Selection of UPR target genes based on the response elements and transcriptional regulation by XBP1 and ATF6

Name	RefSeq	Response elements	Position to TSS	Main UPR TF	Secondary UPR TF
CANX	NM_001024649.1	ERSE	− 109	XBP1	
DNAJB9	NM_012328.2	CRE-like ACGT core	− 1955 − 59	XBP1	
DNAJC10	NM_001271581.1	UPRE	− 132	XBP1	
ERLEC1	NM_015701.4	ERSE-26	− 255	XBP1	
P4HB	NM_000918.3	ACGT core	− 490	XBP1	
SYVN1	NM_032431.2	UPRE ERSE	− 557 − 521	XBP1	
VCP	NM_007126.3	URPE ACGT core ERSE-26	− 452 − 18,167 + 1342	XBP1	
DNAJB11	NM_016306.5	3× ERSE ACGT core	− 357 − 2238	XBP1, ATF6	
DNAJC3	NM_006260.4	ERSE	− 233	XBP1, ATF6	
EDEM1	NM_014674.2	ERSE	− 411	XBP1, ATF6	
HYOU1	NM_006389.4	UPRE	− 242	XBP1, ATF6	
PDIA3	NM_005313.4	ERSE UPRE	− 159 − 4056	XBP1, ATF6	
PDIA6	NM_001282704.1	ERSE	− 157	XBP1, ATF6	
XBP1	NM_005080.3	ERSE	− 78	XBP1, ATF6	
HERPUD1	NM_014685.3	ERSE-II ERSE ACGT core	− 167 − 209 − 563	ATF6	XBP1
HSPA5	NM_005347.4	3× ERSE ERSE-II	− 68 − 107	ATF6	XBP1
PDIA4	NM_004911.4	ERSE UPRE	− 12,331 + 6829	ATF6	XBP1
OS9	NM_006812.3	ACGT core ERSE	− 601 + 328	AFT6	XBP1
SEL1L	NM_005065.5	–	–	AFT6	
SIL1	NM_001037633.1	–	–	–	

The selected IRE1α-XBP1 target genes were characterised based on different parameters. Two parameters are reported: (i) the presence of ERSE elements in their promoter regions, covering 20 kb upstream and 5 kb downstream of ATG, and (ii) evidence for their expression regulation by XBP1 or ATF6 (references detailed are reported in Table S4). In addition, other parameters were considered for the XBP1 target gene selection such as (iii) function, defined by biological process through gene ontology analysis (see Fig. S1, Table S5); (iv) intra-cellular localisation, obtained by different databases (see “Material and methods”); and (v) the association with neurodegenerative diseases, in particular with ALS, AD and FTLD (see Table S4). Among the 18 target genes selected, 7 genes are regulated exclusively by XBP1, 1 exclusively by ATF6 and 10 regulated by both TFs. In addition, one gene (SIL1) was selected because it is not regulated by neither XBP1 nor ATF6

*ERSE* ER stress response element, *UPRE* unfolded protein response element

### The IRE1α-XBP1 pathway is activated in ALS and AD but not in FTLD cases

**Fig. 2:**
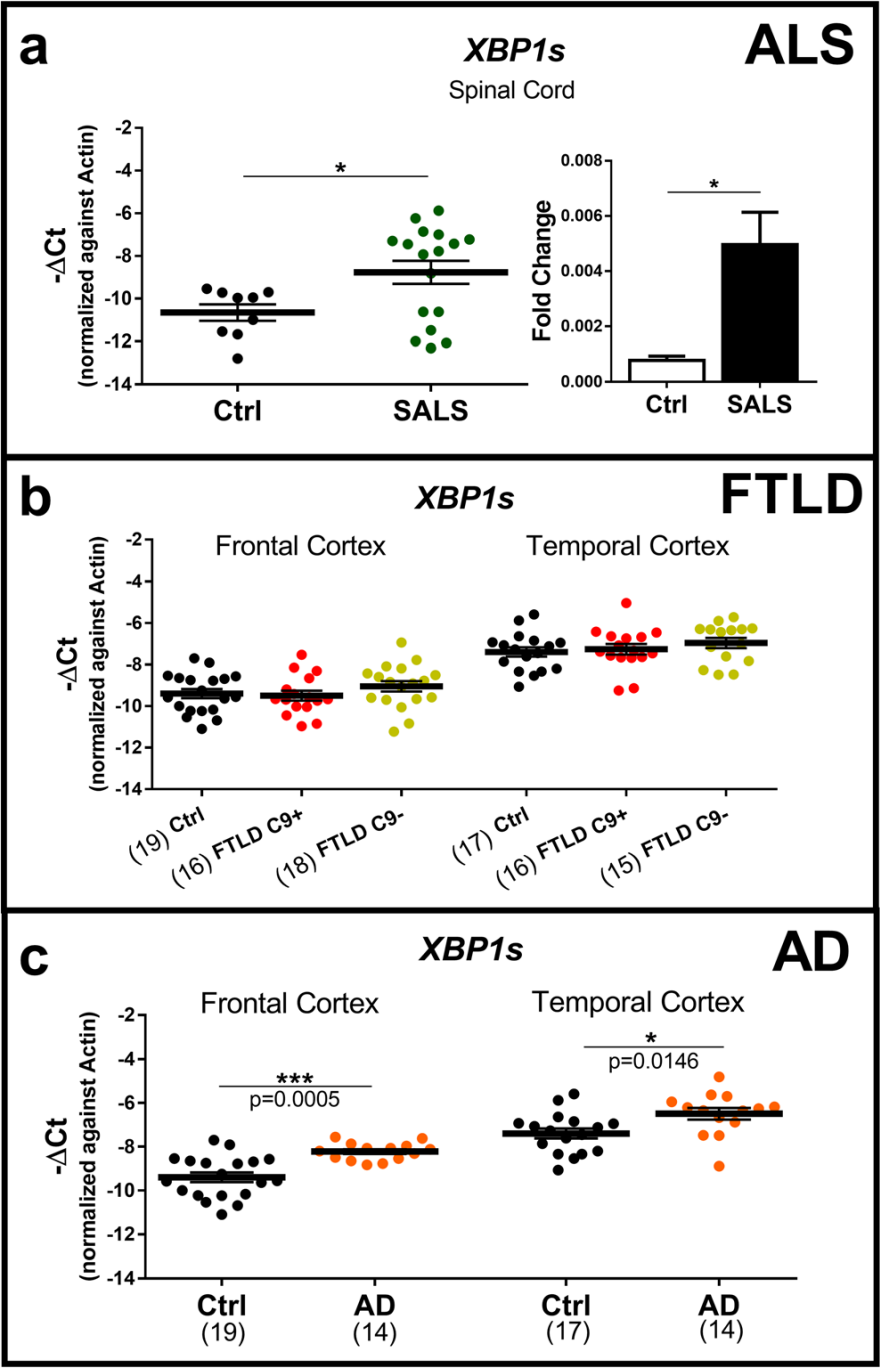
Gene expression analysis of the spliced form of XBP1 (XBP1s) in the spinal cord and frontal and temporal cortex of amyotrophic lateral sclerosis (ALS), Alzheimer’s disease (AD) and frontotemporal lobar dementia (FTLD) cases. **a** mRNA expression analysis of XBP1s in the spinal cord of healthy individuals (Ctrl, grey) and cases of amyotrophic lateral sclerosis (SALS, dark green). Scatter plots and bar plots are representations for the same samples. Ctrl, *n* = 9; ALS, *n* = 17. **b** mRNA expression analysis of XBP1s in the frontal and temporal cortex of healthy individuals (Ctrl, grey) and cases of Alzheimer’s disease (AD, orange). In the frontal cortex, Ctrl, *n* = 19; AD, *n* = 14. In the temporal cortex, Ctrl, *n* = 17; AD, *n* = 14. **c** Gene expression analysis of the spliced form of XBP1 (XBP1s) in the frontal and temporal cortex of healthy individuals (Ctrl, grey) and patients with frontotemporal lobar dementia with (FTLD C9+, red) and without (FTLD C9−, green) C9orf72 repeat expansions. In the frontal cortex, Ctrl, *n* = 19; FTLD C9+, *n* = 16; FTLD C9−, *n* = 18. In the temporal cortex, Ctrl, *n* = 17; FTLD C9+, *n* = 16; FTLD C9−, *n* = 15. Means and SEMs were used to represent the data. The dots represent the samples analysed. According to the D’Agostino and Pearson normality test, all data are normally distributed. The unpaired *t* test or two-way ANOVA, followed by Tukey’s multiple comparison tests, was used. *P < 0.05; **P < 0.01, ***P < 0.001. ALS, amyotrophic lateral sclerosis; AD, Alzheimer’s disease; FTLD, frontotemporal lobar dementia; Ctrl, control

Initially, we investigated the activation of the IRE1α-XBP1 pathway in ALS, AD and FTLD cases. To this end, we analysed the expression of the spliced form of XBP1 (XBP1s) in the different brain regions associated with each disease. In agreement with the literature (Prell et al. 2012), we found a substantial increase in XBP1s expression in the spinal cord derived from sporadic cases of ALS (Fig. 2a). Frozen tissues from frontal and temporal cortex regions (Lindberg et al. 2012; Möller et al. 2016) were used to assess the IRE1α-XBP1 pathway activation in AD and FTLD cases. The FTLD cases, all neuropathologically characterised by TDP-43 inclusions, were divided in two groups based on the presence (FTLD C9+) or absence (FTLD C9−) of *C9orf72* repeat expansions. *C9orf72* repeat expansions are positively correlated with the risk of familial and SALS and are recognised as the most common genetic cause of ALS and FTLD (Boylan 2015). Although XBP1s was found to be upregulated in ALS, no changes in XBP1s expression were detected either in *C9orf72*-positive or *C9orf72*-negative FTLD cases (Fig. 2b). Accordingly, the expression of XBP1 target genes such as *HSPA5* and *DNAJB9* was found to be comparable between FTLD and control cases (additional file: Fig. S2A). However, we found a significant upregulation of XBP1s in both frontal and temporal cortex regions of AD cases (Fig. 2c). These results demonstrated that the IRE1α-XBP1 pathway is activated in ALS and AD cases but not in FTLD cases (whether or not a *C9orf72* hexanucleotide repeat expansion was present).

### Characterisation of the expression of XBP1 and ATF6 target genes in the spinal cord of amyotrophic lateral sclerosis cases

**Fig. 3:**
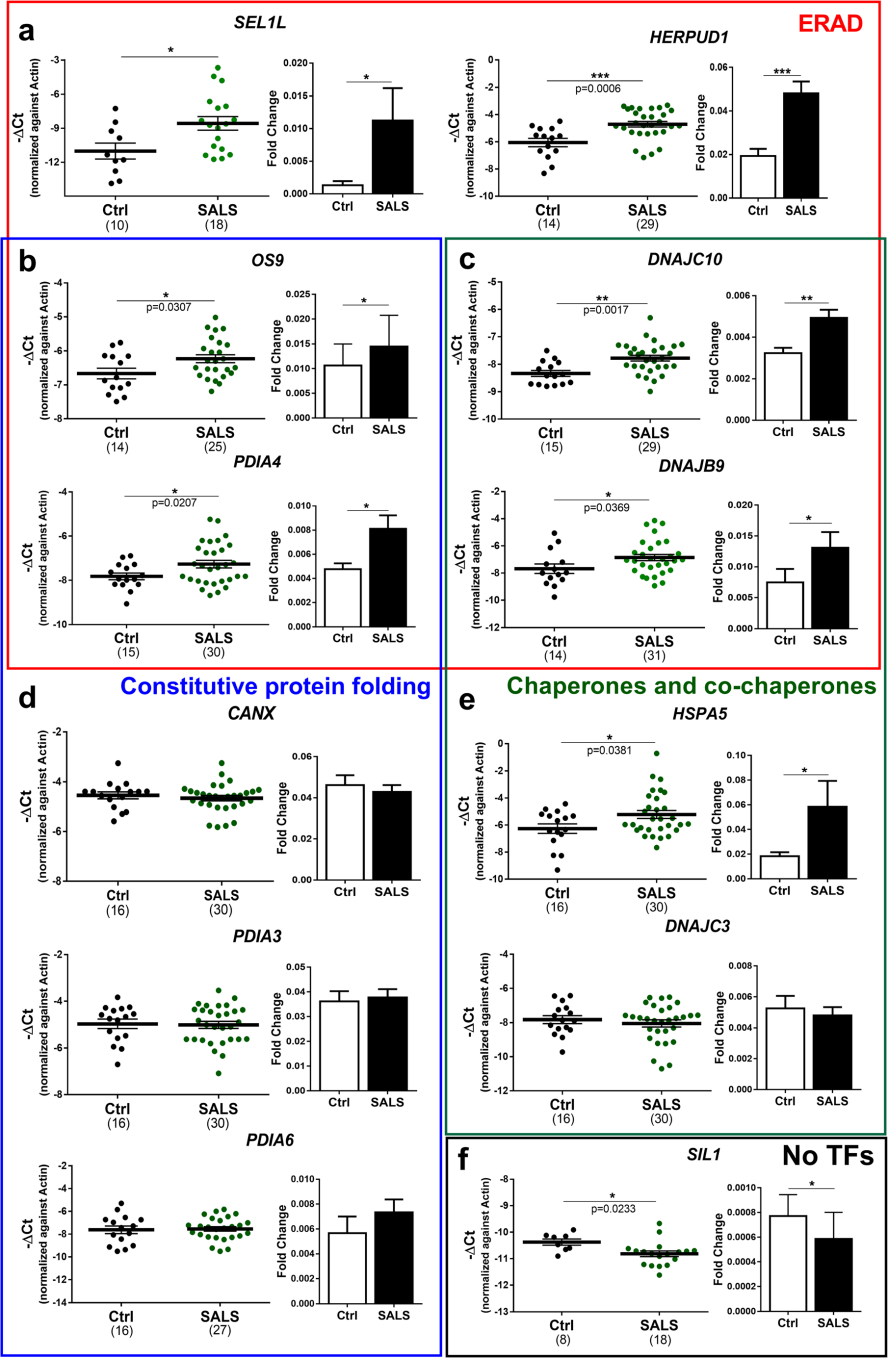
Gene expression of XBP1 target genes tested in the spinal cord derived from sporadic cases of ALS (SALS). **a**–**e** Gene expression of a representative group of XBP1 and ATF6 target genes in spinal cord post-mortem samples derived from healthy individuals (Ctrl, black) and cases of amyotrophic lateral sclerosis (SALS, dark green). Genes were grouped by their main function in ERAD, *endoplasmic reticulum-associated degradation (outlined in red*), *constitutive protein folding and chaperones (outlined in blue) and co-chaperones (outlined in green)*. **f** Gene expression of a gene (SIL1) which is not regulated either by XBP1 or ATF6. Means and SEMs were used to represent the data. The dots represent individual samples and the numbers under the graphs represent the number of samples analysed. SALS, sporadic amyotrophic lateral sclerosis; Ctrl, control; No TFs, not regulated by transcription factors (XBP1 and/or ATF6). According to the D’Agostino and Pearson normality test, all data are normally distributed. The unpaired *t* test was used; *P < 0.05; **P < 0.01, ***P < 0.001

The activation of the IRE1α-XBP1, together with the ATF6 pathway, was investigated through the expression of the selected 19 target genes (Table 1) in spinal cord samples derived from healthy individuals and sporadic cases of ALS (SALS). These genes were grouped in functional clusters as *constitutive protein folding*, *chaperones and co-chaperones*, and *endoplasmic reticulum-associated degradation* (*ERAD*) based on their main function (Fig. 3)*.* The expression of almost all the genes involved in ERAD such as *HERPUD1*, *SEL1L*, *OS9* and *PDIA4* was increased in SALS cases (Fig. 3a, b**)**. The upregulation of *SEL1L*, another gene implicated in protein degradation processes, indicates a likely activation of ATF6 (Yamamoto et al. 2007) (Fig. 3b) while the activation of the IRE1α-XBP1 pathway was confirmed by the increase in the expression of *DNAJB9* and *DNAJC10* genes (Fig. 3c). Protein-protein interaction and gene ontology analysis revealed that the upregulated genes in ALS cases were part of a close network functionally enriched in *ERAD* and *ubiquitin-dependent protein catabolic processes* terms (additional file: Fig. S3B-S3C).

Conversely, genes involved in the early stages of the *constitutive protein folding* processes such as *CANX*, *PDIA3* and *PDIA6* were not differentially expressed in ALS (Fig. 3d). Co-chaperones involved in the folding processes such as DNAJC3 and DNAJB11 (Shoulders et al. 2013) showed comparable expression levels between control and SALS cases (Fig. 3e, additional file: Fig. S3A). In the *chaperones and co-chaperones* cluster, however, we found an elevated expression of *HSPA5* gene, which encodes for a well-established marker of ER stress (BiP) and is regulated to the same extent by both TFs (Prell et al. 2012) (Fig. 3e). Notably, we found a decrease in the expression of *SIL1*, a gene that is not regulated by either XBP1 or ATF6 and that encodes a nucleotide exchange factor for the ER chaperone HSPA5 (Fig. 3f).

Activated IRE1α is also involved in the cleavage of metabolic mRNAs through a process known as IRE1α-dependent decay (RIDD) (Maurel et al. 2014; Tam et al. 2014; Hetz and Saxena 2017). RIDD is a pleiotropic mechanism affecting the expression of different sets of genes in different tissues, and the assessment of its role in neurodegenerative disease remains almost unknown. The major RIDD substrates identified in human and mouse are SCARA3 (Maurel et al. 2014; Tam et al. 2014), SPARC (Maurel et al. 2014; Tam et al. 2014) and BLOC1S1 (Maurel et al. 2014; Bright et al. 2015). In particular, the latter has been hypothesised to be a possible universal RIDD target (Bright et al. 2015). Thus, we quantified the mRNAs of these genes in order to assess whether the RIDD activity of IRE1α is active in human post-mortem tissues. However, none of these genes showed a downregulation in the spinal cord of ALS (additional file: Fig. S2B). These results indicate that ATF6 and IRE1α pathways activate a set of genes mainly involved in ERAD and protein degradation processes.

### Characterisation of the expression of XBP1 target genes in the frontal and temporal cortex of cases of Alzheimer’s disease

**Fig. 4:**
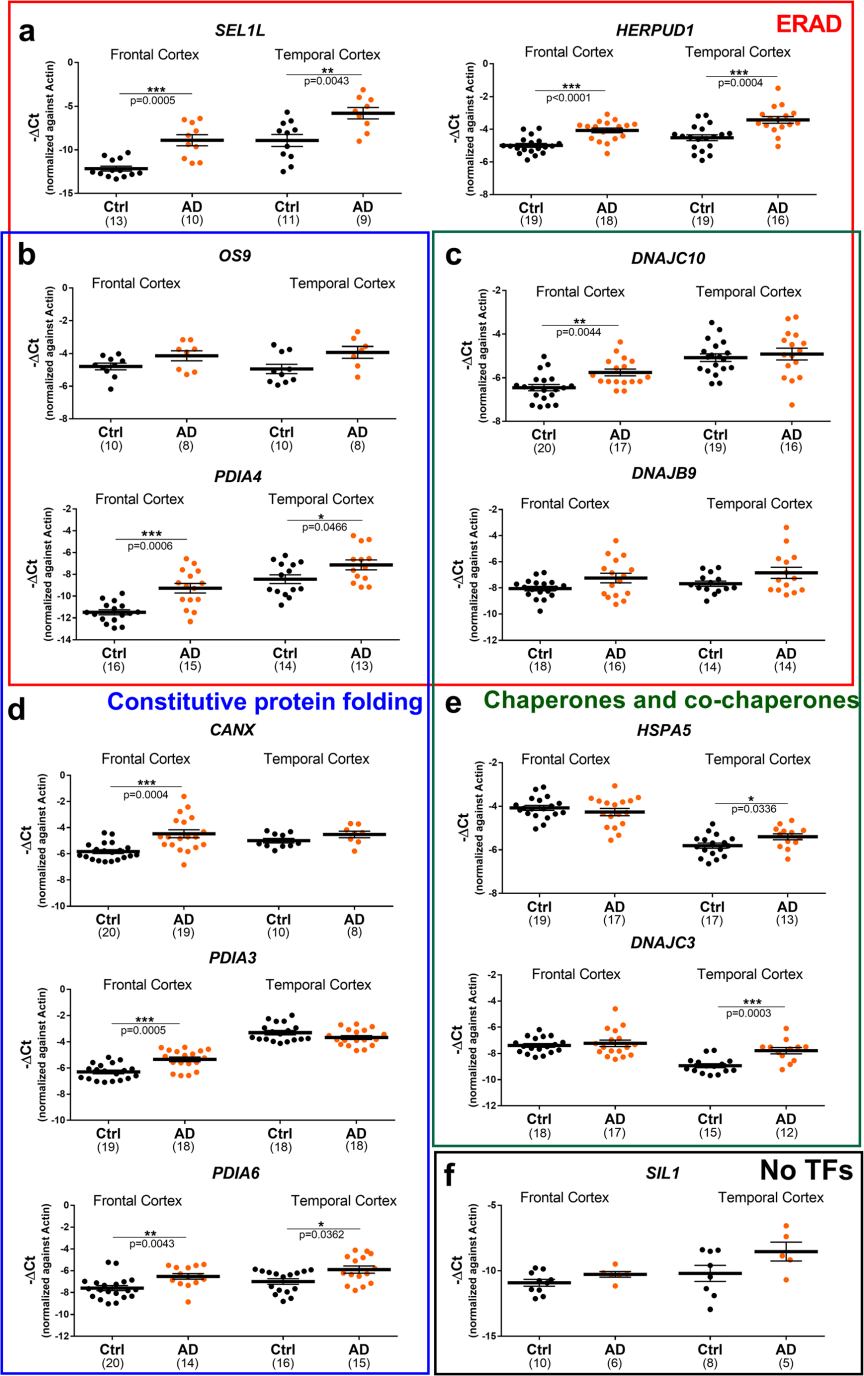
Gene expression analysis of XBP1 target genes tested in the frontal and temporal cortex of the brain derived from Alzheimer’s disease (AD) cases. **a**–**e** Gene expression of a representative group of XBP1 and ATF6 target genes in frontal and temporal cortex post-mortem samples derived from healthy individuals (Ctrl, grey) and Alzheimer’s disease cases (AD, orange). Genes were grouped by their main function. **f** Gene expression of a gene (SIL1) which is not regulated either by XBP1 or ATF6. Means and SEMs were used to represent the data. The dots represent the individual samples and the numbers under the graphs represent the number of samples analysed. AD, Alzheimer’s disease; Ctrl, control; No TFs, not regulated by transcription factors (XBP1 and/or ATF6). According to the D’Agostino and Pearson normality test, all data are normally distributed. The unpaired *t* test was used; *P < 0.05; **P < 0.01, ***P < 0.001

After establishing that a significant increase occurred in XBP1s levels (Fig. 2b), we investigated the expression of the selected 19 genes in frontal and temporal regions of the human brain derived from AD cases. Many of the genes upregulated in ALS that are involved in ERAD such as *HERPUD1*, *SEL1L*, *PDIA4* and *DNAJC10* also showed an increased expression in one or both cortical regions of AD cases (Fig. 4a, d). In contrast, the expression of *OS9* and *DNAJB9*, upregulated in SALS cases, was comparable between disease and control cases (Fig. 4b, c). Another remarkable difference between AD and ALS cases was that the expression of protein disulphide isomerase (PDI) family members involved in protein folding processes such as *PDIA6* and *PDIA3*, were selectively upregulated in AD (Fig. 4d) but not in ALS. Interestingly, a difference in gene expression also emerged between the cortical regions of AD cases. *PDIA3* and *CANX*, two genes involved in the lectin folding cycle, were upregulated in the frontal cortex (Fig. 4d) while the expression of *HSPA5* and its co-chaperone *DNAJC3* (Shoulders et al. 2013), involved in protein folding processes, was increased in the temporal cortex (Fig. 4e). *ERLEC1* was also differently expressed in temporal compared to the frontal cortex where it has been found to be downregulated (additional file: Fig. 4SA).

The relevance of protein folding processes in AD was further confirmed by GO analysis of the upregulated genes in the frontal and temporal cortex, which showed a functional enrichment in *endoplasmic reticulum unfolded protein response* and *protein folding in endoplasmic reticulum stress* terms (additional file: Fig. S4B-C). Similarly to ALS, none of the RIDD targets showed a decrease in expression in AD cases, but surprisingly, the expression of *BLOC1S1* gene was found to be increased in the frontal cortex (additional file: Fig. S2C). These results suggest a strong involvement of IRE1α-XBP1 and ATF6 pathways, which induce the expression of genes involved in both protein degradation and protein folding such as PDI family members in AD.

### Protein level analysis of representative genes upregulated or unchanged in the spinal cord and temporal cortex

Western blot (WB) analysis was carried out on a subset of proteins in the spinal cord and temporal cortex including genes that were both upregulated or unchanged in order to substantiate changes found in mRNA. In the spinal cord, we analysed four proteins, three upregulated (*HSPA5*, *DNAJC10*, *HERPUD1*) and one unchanged (*PDIA6*). Indeed, WB results were consistent with these findings. The expression of all three upregulated genes showed a substantial increase also at the protein level (Fig. 6a, b) while, again consistent with qPCR results, PDIA6 showed no change. The same proteins were investigated in the temporal cortex. Although fewer samples were analysed, WB results were consistent with the qPCR findings also in this cortical region. The expression of the three upregulated genes (*HSPA5*, *HERPUD1*, *PDIA6*) and *DNAJC10*, which was unchanged at the mRNA level, showed a similar trend at the protein level. These results demonstrate that genes, which were upregulated in the spinal cord and temporal cortex of ALS and AD cases, show an increase also at the protein level.

## Discussion

The UPR is a network of signalling pathways that respond to ER stress. Among the UPR signalling pathways, IRE1α and ATF6 predominantly play a pro-survival role through the transactivation of the basic-leucine zipper (bZIP) transcription factors, XBP1 and ATF6 (Yoshida et al. 2001; Adachi et al. 2008). In this study, we found that IRE1α-XBP1 and ATF6 pathways activate different sets of genes in the spinal cord of SALS compared to frontal and temporal cortex of AD suggesting that different mechanisms of UPR activation are involved.

**Fig. 5:**
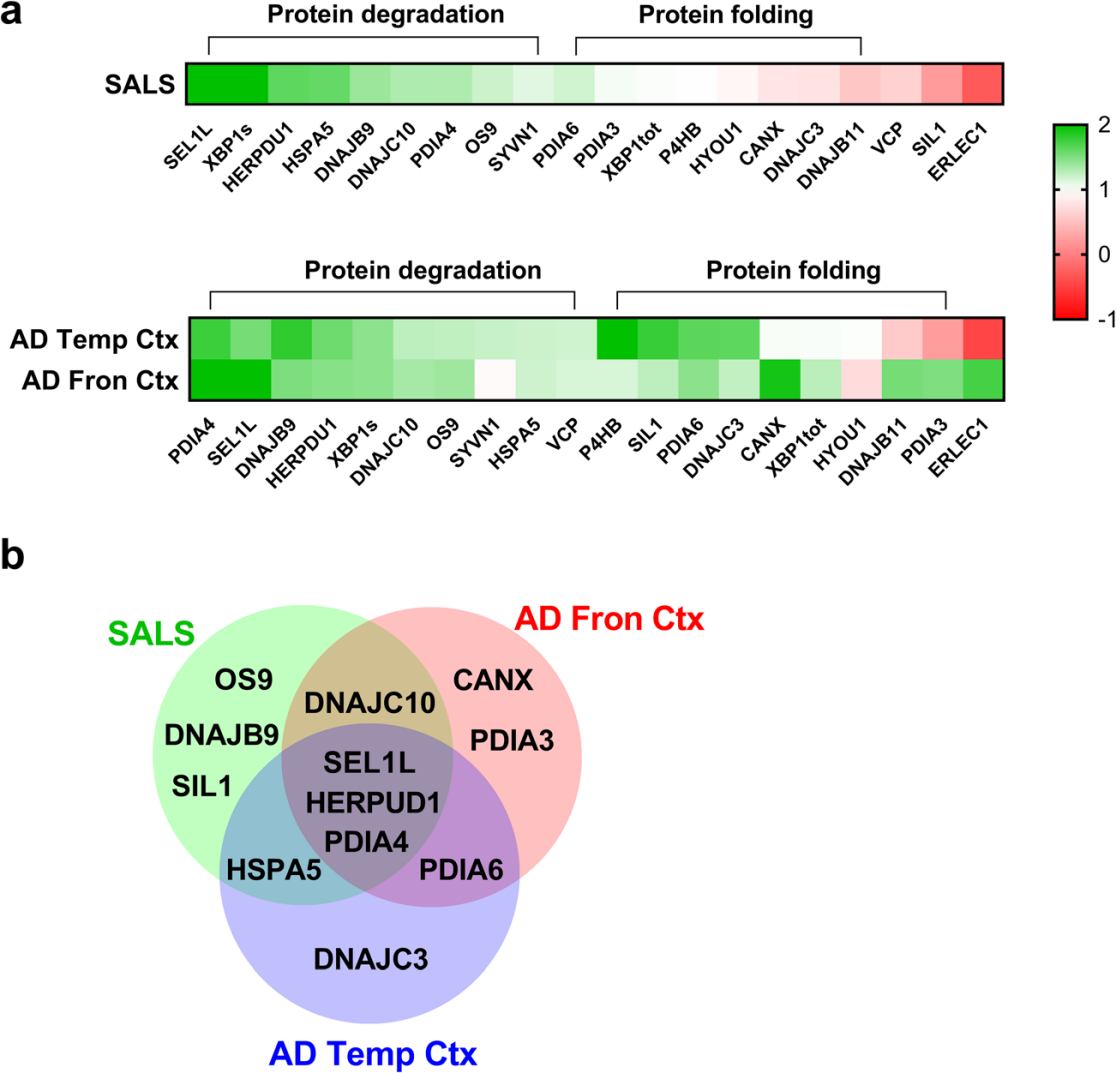
Comparison between gene expression profiles in SALS and frontal and temporal cortex of AD cases. **a** Heat map of the gene expression levels of the 20 UPR target genes regulated by ATF6 and XBP1 and analysed in the lumbar spinal cord of SALS cases and the frontal and temporal cortex of AD cases. The gene expression was normalised against the controls and log2 FC was plotted. Each row in the heat maps corresponds to a tissue analysed; each column corresponds to a specific gene. The coloured bar indicates the range of intensity values for each gene in the heat maps. **b** Venn diagram for the upregulated genes found in SALS cases and AD cases. SALS, sporadic amyotrophic lateral sclerosis; AD temp Ctx, Alzheimer’s disease temporal cortex; AD Fron Ctx, Alzheimer’s disease frontal cortex

**Fig. 6:**
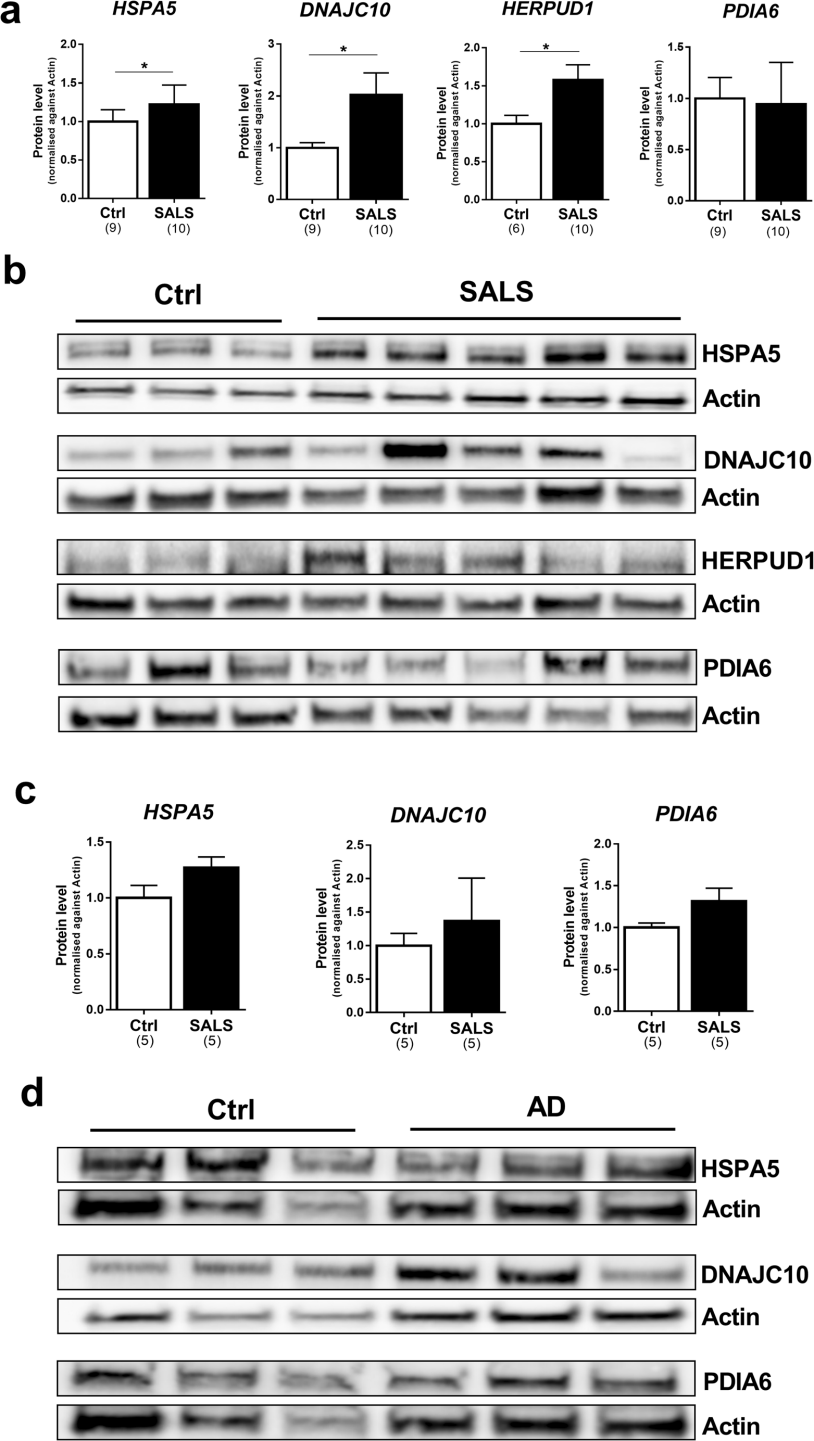
Protein level analysis of representative genes upregulated or unchanged in the spinal cord and temporal cortex. **a** Western blot analyses are shown for HSPA5, DNAJC10, HREPUD1 and PDIA6 detected in the spinal cord of healthy individuals (Ctrl) and SALS cases (SALS). Quantitative analyses of each protein were performed by densitometry of the relative bands in control and SALS cases. Values were obtained using actin as a reference gene. Means and SEMs were used to represent the data. The numbers under the graphs represent the number of samples analysed. SALS, sporadic amyotrophic lateral sclerosis; Ctrl, control. According to the D’Agostino and Pearson normality test, all data are normally distributed. The unpaired *t* test was used; *P < 0.05. **b** Representative western blots are shown for HSPA5, DNAJC10, HREPUD1 and PDIA6. **c** Western blot analyses are shown for HSPA5, DNAJC10 and PDIA6 detected in the temporal cortex of healthy induvial (Ctrl) and AD cases (AD). Quantitative analyses of each protein were performed by densitometry of the relative bands in control and SALS cases. Values were obtained using actin as a reference gene. Means and SEMs were used to represent the data. The numbers under the graphs represent the number of samples analysed. AD, Alzheimer’s disease; Ctrl, control. The unpaired *t* test was used; *P < 0.05. **d** Representative western blots are shown for HSPA5, DNAJC10 and PDIA6

In the spinal cord of ALS cases, we found a substantial increase in the expression of genes associated with ERAD. ERAD fulfils a crucial function through the clearance of misfolded proteins from the lumen of ER and, when impaired, results in accumulation of unfolded proteins (Redler and Dokholyan 2012). The relevance of this process in ALS is further confirmed by the fact that several disease-causative mutations occur in genes involved in (e.g. *VCP*) or affecting (e.g. *SOD1*) these pathways (Boylan 2015). In fact, mutant SOD1 protein interacts specifically with Derlin-1, a component of ERAD machinery, and triggers ER stress-induced apoptosis in motor neurons through dysfunction of ERAD (Nishitoh et al. 2008). We extended the investigation to other ERAD components and we found that the expression of two constitutive members of the ubiquin-degradation machinery, *HERPUD1* and *SEL1L*, as well as two genes associated with ERAD, *PDIA4* and *OS9*, was increased in human spinal cord of SALS cases. (Fig. 5a, b). In addition, we discovered that the expression of two co-chaperones, *DNAJB9* and *DNAJC10*, was increased in disease cases. These co-chaperones belong to the DNAJ family, which are proteins that can modulate the activity of HSPA5/BiP, the only HSP70 chaperone present in the ER (Kampinga and Bergink 2016). Notably, DNAJB9 and DNAJC10 are known to be associated with ERAD (Behnke et al. 2015) suggesting that their interaction with HSPA5/BiP promotes protein degradation processes and suppresses the cell death induced by ER stress (Kurisu et al. 2003). This is further reinforced by the confirmation of the upregulation of HSPA5/BiP, already shown by Atkin et al. (Atkin et al. 2008), and DNAJC10 at the protein level in the spinal cord of SALS cases (Fig. 6). These results, in addition to the upregulation of HERPUD1 protein, indicate that the degradation machinery is upregulated at both mRNA and protein levels. Overloading of ERAD results in accumulation of unfolded proteins leading the activation of the UPR. In this context, changes in the expression of *SEL1L*, a gene regulated exclusively by ATF6 (Kaneko et al. 2007), and *DNAJB9*, a gene regulated exclusively by XBP1 [3, 6, 49], suggest that these two UPR pathways are co-activated in SALS cases. Interestingly, the only downregulation observed in ALS cases was seen in the expression of *SIL1*, a gene that is not regulated by either ATF6 or XBP1. The *SIL1* gene encodes for a nucleotide exchange factor associated with Hsp70 that facilitates substrate release from BiP by stimulating the release of ADP (Behnke et al. 2015). Recently, de L’Etang et al. showed that SIL1 levels were selectively reduced in vulnerable motor neurons of *SOD1-G93A* mice (L’Etang et al. 2015). Thus, the reduced expression of *SIL1*, detected for the first time in human samples, could reflect the specific loss of motor neurons in the spinal cord of ALS cases.

A different profile of gene regulation was found in AD whereby the upregulated genes were predominantly involved in multiple stages of protein homeostasis (Fig. 5a, b). As occurs in ALS, we found an increased expression of ERAD genes such as *PDIA4*, *HERPUD1*, *DNAJC10* and *SEL1L* in frontal and/or in temporal cortical regions of AD cases. Besides ERAD, we also found a profound activation of genes involved in protein folding processes. One example is given by the increase of *HSPA5* expression in the temporal cortex which is associated with the upregulation of DNAJC3, a co-chaperone involved in early stage of protein folding (Shoulders et al. 2013; Behnke et al. 2015). In ALS, conversely, *HSPA5* showed a co-upregulation with DNAJs involved in protein degradation, a disease difference worthy of further investigation. Another remarkable difference between gene expression profiles in these diseases is highlighted by the strong increase in the expression of several PDI family members in AD cases. Elevated expression of *PDIA6*, *PDIA3*, *PDIA4* and *DNAJC10* (also known as PDIA19) emphasises the key role of PDI family members in cortical regions. PDI family members play a crucial role in oxidative folding and chaperone-mediated quality control of proteins by acting as a general response when misfolded proteins start to accumulate in the ER (Benham 2012).

Distinct gene expression profiles emerged also between the two cortical regions analysed. Genes encoding for proteins involved in the ER lectin cycle such as *CANX* and *PDIA3* were upregulated in the frontal cortex, while genes involved in chaperone and co-chaperone activity, such as *HSPA5* and *DNAJC3*, were upregulated in the temporal cortex (Fig. 5a, b). Although both regions are characterised by changes in protein homeostasis during AD pathology (Hoozemans et al. 2005; Lindberg et al. 2012; Möller et al. 2016), our results indicate that either specific responses to ER stress are activated in the frontal and temporal cortex or that the different regions reflect a different stage in disease progression. Moreover, this evidence suggests that brain-region-specific factors must play a role in determining selective vulnerability such as the abundance of components of the protein folding and quality control systems (Asuni et al. 2013; Jackson 2014). Notably, the majority of the changes in gene expression occurred in the regions where the genes were expressed at lower levels (Fig. 4). Moreover, HSPA5, DNAJC10 and PDIA6 showed a trend to increase at the protein level confirming the gene expression results. In terms of magnitude of expression changes, the most robust upregulations were seen for *SEL1L* and *HERPUD1* in both AD and ALS indicating a possible common mechanism of action. Finally, the dysregulated expression of genes regulated by XBP1 and ATF6 indicates that both ATF6 and IRE1α-XBP1 pathways are activated in both cortical regions of AD cases. The upregulation of *HERPUD1* and *HSPA5*, common genes regulated by all three UPR pathways (Fig. 1a), could also reflect an activation of the PERK pathway. Although the PERK pathway has been found to be involved in AD (Rozpedek et al. 2015; Yang et al. 2016), in this study, we focused on specific responses activated by IRE1α-XBP1 and ATF6 pathways which share several target genes. In contrast the PERK pathway showed little overlap with the other UPR pathways (additional file: Fig. S1B-D). When activated, IRE1α is able to process XBP1 as well as other mRNAs though a process called IRE1α-dependent decay (RIDD) (Maurel et al. 2014; Tam et al. 2014; Hetz and Saxena 2017). Although it has been proposed that RIDD can contribute to cell death during chronic conditions of ER stress (Maurel et al. 2014), the physiological role of this process in neurodegenerative disease is still unclear. We found that the expression of the major RIDD substrates was not altered in either ALS or AD. Although further investigations need to be performed in order to understand the role of RIDD in neurodegenerative diseases, we concluded that the RNase activity of IRE1α does not affect the mRNA level of the established RIDD targets in ALS and AD. Surprisingly, we found that one of the putative RIDD target, *BLOC1S1*, was profoundly upregulated in the frontal cortex of AD (additional file: Fig. S2B). This upregulation could indicate an activation of UPR downstream pathways since BLOC1S1, in addition to being an RIDD substrate, is also involved in lysosomal biogenesis (Pu et al. 2015)

Several studies have demonstrated cooperation between ATF6 and IRE1α-XBP1 pathways (Yamamoto et al. 2007; Shoulders et al. 2013), and this is also confirmed by the partial overlap of the biological processes associated with the target genes regulated by these two (Fig. 1a, additional file: S1B). In this context, there is evidence suggesting that the cooperative effect of ATF6 and XBP1 is due to their ability to form heterodimers which have an increased affinity for the binding to specific *cis*-acting elements localised in the promoter regions of major UPR genes (Newman 2003; Yamamoto et al. 2007; Shoulders et al. 2013). We showed that the majority of the genes that contain ERSE and ERSE-II response elements in their promoters are regulated by both transcription factors, while ACGT core and ERSE-26 elements appear to be regulated specifically by XBP1 (Fig. 1d). Notably, these ERSE elements are not a direct binding site of ATF4, the main transcription factor activated by the PERK pathway (Ma et al. 2002). In addition, the UPRE consensus sequence appears to be associated with both TFs although a larger number of candidates need to be studied before a more robust conclusion can be reached. Further investigations are also necessary to assess the functional role of these consensus sequences in the regulation of each gene.

An interesting point of view was elaborated by Mori et al. who proposed a model whereby the ATF6-mediated response is unidirectional (refolding only) while it is shifted by a XBP1-mediated response to a bidirectional mode (refolding plus degradation) depending on the nature of the stimulus (Yoshida et al. 2003; Yamamoto et al. 2007). This model is also supported by the fact that ATF6 alone is still able to induce the transcription of ER chaperones but not of ERAD components (Yamamoto et al. 2007). Thus, the substantial upregulation of genes found in ALS cases could indicate that the cooperation between ATF6α and XBP1 plays a crucial role in the pathophysiological mechanism of this disease. In AD, instead, the cooperative effect between these two pathways is less evident probably due to a strong contribution of each of these pathways.

## Conclusions

In this study, we have compared the ER homeostatic pathways IRE1α-XBP1 and ATF6 in different neurodegenerative disorders. Specifically, our results reveal that disease-specific patterns of IRE1α-XBP1 and ATF6 target genes are activated in AD and ALS. The identification of these patterns provides a valuable source of information from which to develop new selective therapeutic strategies in both ALS and AD. Moreover, since UPR activation has been proposed to be activated in early stages of ALS and AD, the differential patterns of gene activation in these disorders can be used as potential early biomarkers.

## Electronic supplementary material

ESM 1 (PDF 4337 kb)

## Abbreviations

AD: Alzheimer’s disease
ALS: Amyotrophic lateral sclerosis
ATF4: Activating transcription factor 4
ATF6: Activating transcription factor 6
BiP: Binding immunoglobulin protein
DNAJs: DnaJ heat shock protein family
ER: Endoplasmic reticulum
ERAD: ER-associated degradation
ERSE: ER stress response elements
FTLD: Frontotemporal lobar degeneration
IRE1: Inositol-requiring enzyme 1
NFE2L2: Nuclear factor, erythroid 2-like 2
PDI: Protein disulphide isomerase
PERK: Protein kinase RNA-like endoplasmic reticulum kinase
PS1: Presenilin-1
qPCR: Quantitative polymerase chain reaction
TF: Transcription factor
UPR: Unfolded protein response
UPRE: Unfolded protein response element
XBP1: X box-binding protein 1
