# Supplementary material for: Amyotrophic lateral sclerosis (ALS) and Alzheimer’s disease (AD) are characterised by differential activation of ER stress pathways: focus on UPR target genes
Source: Cell Stress Chaperones. 2018 May 4;23(5):897–912. doi: 10.1007/s12192-018-0897-y (PMC6111088; doi:10.1007/s12192-018-0897-y)
Supplement: Supplementary file 1 — (PDF 4337 kb) [file 12192_2018_897_MOESM1_ESM.pdf]

Supporting Information

SI Material and Methods

Tissue samples

The study was approved by the Riverside Research Ethics Committee and was carried out according to their guidelines. Diagnosis of ALS was confirmed by El Escorial diagnostic criteria defined by the World Federation of Neurology (Brooks, 1994; Brooks et al., 2000).

Subjects

Frozen dorsolateral prefrontal cortex (PFC) and temporal cortex (TCtx) tissue were obtained from 79 case subjects. Controls had no neuropathological signs aside from age-related changes, Alzheimer's disease samples (AD) were identified by Braak hyperphosphorylated tau stage 5 and above, while frontotemporal lobar degeneration samples were identified by presence of TDP43-positive inclusions. FTLD cases were genetically screened for C9orf72 mutations and sorted into C9orf72 mutation-positive (FTLD C9+) or -negative (FTLD C9-). Table S5 summarises the sex, age at death, post-mortem delay, and clinical details of the patient samples. There was no significant difference between groups and controls, except for an earlier age at death in the C9orf72+ FTLD group, which is typical of the illness (Gijselinck et al., 2016).

Selection of gene targets of UPR Transcription Factors

To identify the gene candidates for our study, we performed *in silico* systematic analysis on the target genes of UPR Transcription Factors (TFs) such as XBP1, ATF6 and ATF4/NRF2 (Cullinan et al., 2003; Fels and Koumenis, 2006; Yoshida et al., 2001). Three different databases (JASPAR, Reactome and TRUSST) were used to extract the names of the genes regulated by the UPR TFs (Croft et al., 2011; Han et al., 2015; Sandelin et al., 2004) selecting *Homo Sapiens* genome 19 (hg19) as background model. Successively the XBP1 targets genes were screened for their tissue expression (The Human Protein Atlas, Human Allen brain Atlas and Expression Atlas (Petryszak et al., 2016), http://www.proteinatlas.org) and only the genes expressed in CNS were selected.

Bioinformatics

The genes extracted from the TF databases were functionally annotated using the Database for Annotation, Visualisation and Integrated Discovery (DAVID) Bioinformatics Resources 6.8 (Huang et al., 2009, 2008). Gene ontology (GO) was used to classify these genes and their relative proteins, including their involvement in biological processes (BP), as cellular compartments (CC) and their molecular function (MF). Homo sapiens whole genome was used as background. The web application for the automated integrative analysis BioInfoMiner program© (bioinfominer.com) by e-NIOS Company was used to perform the reduction of GO terms redundancy and annotation bias.

Cis-Acting Elements Analysis

In addition to cis-Acting elements analysis, we merged the resulting list of candidates with a human and murine gene lists based on the presence of ER stress response elements published in literature (Acosta-Alvear

et al., 2007; Dombroski et al., 2010).

RNA extraction and cDNA synthesis

Purity and concentration of RNA was quantified using a NanoDrop spectrophotometer. The RNA obtained was retro-transcribed in cDNA using cloned an Avian Myeloblastosis Virus (AMV) cDNA synthesis kit (Invitrogen). Primer3 web software was used to design primers for the amplification of target cDNA sequence. In addition, KiCqStart® SYBR® Green Primers (Sigma) were used. To optimize the primers temperature and gradient PCR were performed and the PCR products were checked on agarose gels. The list of primers is Table S5.

Quantitative real-time PCR (qPCR)

qPCR was performed using the Power Up™ SYBr™ Green Master Mix with 6 μm of primers. Stratagene® MX3000p qPCR system was used with the following cycling conditions: 95 C for 10 minutes, and then 35 cycles of 95 °C for 30 s, Tm °C (of gene of interest) for 30 s, 72 °C for 1 minute, followed by 95 °C for 1 minute, 63 °C for 30 s, and 95 °C for 30 s. Ct values were normalized to housekeeping gene actin.

Western blot analysis

Appropriate volumes of 4x Laemnli SDS sample buffer (v/v, Alfa Aesar) were then added. Homogenised tissues were warmed at 95°C for 5 min, and cleared by 12000 x g centrifugation in a microcentrifuge for 20 min at 4°C. Supernatants were collected and proteins protein concentration was determined using the Bio-Rad protein assay kit. Wqual protein amounts were separated from the different samples by 10% SDS-PAGE and blotted onto nitrocellulose membranes. Transfer efficiency was checked with Ponceau (Sigma) staining. Blots, blocked in 1% milk/PBS-Tween-20 (PBST), were probed with specific antibodies, washed with PBST and then incubated with peroxidase-conjugated secondary antibody. Finally, each membrane was probed to detect β-actin. The final dilutions and incubation times suggested by the manufacturer were used for each antibody. Immunodetection was performed using the ECL reagents (Thermo Scientific). Densitometry quantification of the bands was performed using ImageJ software (National Institute of Health Bethesda, MD, USA).

References

Acosta-Alvear, D., Zhou, Y., Blais, A., Tsikitis, M., Lents, N.H., Arias, C., Lennon, C.J., Kluger, Y., Dynlacht, B.D., 2007. XBP1 Controls Diverse Cell Type- and Condition-Specific Transcriptional Regulatory Networks. Mol. Cell 27, 53–66. doi:10.1016/j.molcel.2007.06.011

Brooks, B.R., 1994. El Escorial World Federation of Neurology criteria for the diagnosis of amyotrophic lateral sclerosis. Subcommittee on Motor Neuron Diseases/Amyotrophic Lateral Sclerosis of the World Federation of Neurology Research Group on Neuromuscular Diseases and the El Escorial "Clinical limits of amyotrophic lateral sclerosis" workshop contributors. J. Neurol. Sci. 124 Suppl, 96–107.

Brooks, B.R., Miller, R.G., Swash, M., Munsat, T.L., World Federation of Neurology Research Group on Motor Neuron Diseases, 2000. El Escorial revisited: revised criteria for the diagnosis of amyotrophic lateral sclerosis. Amyotroph. Lateral Scler. Other Motor Neuron Disord. 1, 293–9.

Croft, D., O'Kelly, G., Wu, G., Haw, R., Gillespie, M., Matthews, L., Caudy, M., Garapati, P., Gopinath, G., Jassal, B.,

Jupe, S., Kalatskaya, I., Mahajan, S., May, B., Ndegwa, N., Schmidt, E., Shamovsky, V., Yung, C., Birney, E., Hermjakob, H., D'Eustachio, P., Stein, L., 2011. Reactome: a database of reactions, pathways and biological processes. Nucleic Acids Res. 39, D691-7. doi:10.1093/nar/gkq1018

Cullinan, S.B., Zhang, D., Hannink, M., Arvisais, E., Kaufman, R.J., Diehl, J.A., 2003. Nrf2 is a direct PERK substrate and effector of PERK-dependent cell survival. Mol. Cell. Biol. 23, 7198–209.

Dombroski, B.A., Nayak, R.R., Ewens, K.G., Ankener, W., Cheung, V.G., Spielman, R.S., 2010. Gene Expression and Genetic Variation in Response to Endoplasmic Reticulum Stress in Human Cells. Am. J. Hum. Genet. 86, 719–729. doi:10.1016/j.ajhg.2010.03.017

Fels, D.R., Koumenis, C., 2006. The PERK/eIF2alpha/ATF4 module of the UPR in hypoxia resistance and tumor growth. Cancer Biol. Ther. 5, 723–8.

Gijselinck, I., Van Mossevelde, S., van der Zee, J., Sieben, A., Engelborghs, S., De Bleecker, J., Ivanoiu, A., Deryck, O., Edbauer, D., Zhang, M., Heeman, B., Bäumer, V., Van den Broeck, M., Mattheijssens, M., Peeters, K., Rogaeva, E., De Jonghe, P., Cras, P., Martin, J.-J., de Deyn, P.P., Cruts, M., Van Broeckhoven, C., 2016. The C9orf72 repeat size correlates with onset age of disease, DNA methylation and transcriptional downregulation of the promoter. Mol. Psychiatry 21, 1112–24. doi:10.1038/mp.2015.159

Han, H., Shim, H., Shin, D., Shim, J.E., Ko, Y., Shin, J., Kim, H., Cho, A., Kim, E., Lee, T., Kim, H., Kim, K., Yang, S., Bae, D., Yun, A., Kim, S., Kim, C.Y., Cho, H.J., Kang, B., Shin, S., Lee, I., Vaquerizas, J.M., Kummerfeld, S.K., Teichmann, S.A., Luscombe, N.M., Marbach, D., Salgado, H., Faith, J.J., Hodges, P.E., McKee, A.H., Davis, B.P., Payne, W.E., Garrels, J.I., Beyer, A., Gallo, S.M., Marbach, D., Essaghir, A., Zhao, F., Xuan, Z., Liu, L., Zhang, M.Q., Bovolenta, L.A., Acencio, M.L., Lemke, N., Montgomery, S.B., Ihmels, J., Banerjee, N., Zhang, M.Q., Ravasi, T., Roach, J.C., Karczewski, K.J., Snyder, M., Altman, R.B., Tatonetti, N.P., Lee, I., Blom, U.M., Wang, P.I., Shim, J.E., Marcotte, E.M., Chatr-Aryamontri, A., Salwinski, L., Prasad, T.S.K., Orchard, S., Licata, L., Turner, B., Shannon, P., Schriml, L.M., Kanehisa, M., Goto, S., Kawashima, S., Okuno, Y., Hattori, M., Ashburner, M., Hall, J.M., Leongamornlert, D., Mocci, E., Comeau, D.C., Chen, L., Wu, G., Ji, H., Wu, G., Ji, H., Barrett, T., Denkert, C., Huynh-Thu, V.A., Irrthum, A., Wehenkel, L., Geurts, P., 2015. TRRUST: a reference database of human transcriptional regulatory interactions. Sci. Rep. 5, 11432. doi:10.1038/srep11432

Huang, D.W., Sherman, B.T., Lempicki, R.A., 2009. Bioinformatics enrichment tools: paths toward the comprehensive functional analysis of large gene lists. Nucleic Acids Res. 37, 1–13. doi:10.1093/nar/gkn923

Huang, D.W., Sherman, B.T., Lempicki, R.A., 2008. Systematic and integrative analysis of large gene lists using DAVID bioinformatics resources. Nat. Protoc. 4, 44–57. doi:10.1038/nprot.2008.211

Petryszak, R., Keays, M., Tang, Y.A., Fonseca, N.A., Barrera, E., Burdett, T., Füllgrabe, A., Fuentes, A.M.-P., Jupp, S., Koskinen, S., Mannion, O., Huerta, L., Megy, K., Snow, C., Williams, E., Barzine, M., Hastings, E., Weisser, H., Wright, J., Jaiswal, P., Huber, W., Choudhary, J., Parkinson, H.E., Brazma, A., 2016. Expression Atlas update--an integrated database of gene and protein expression in humans, animals and plants. Nucleic Acids Res. 44, D746-52. doi:10.1093/nar/gkv1045

Sandelin, A., Alkema, W., Engström, P., Wasserman, W.W., Lenhard, B., 2004. JASPAR: an open-access database for eukaryotic transcription factor binding profiles. Nucleic Acids Res. 32, D91-4. doi:10.1093/nar/gkh012

Yoshida, H., Matsui, T., Yamamoto, A., Okada, T., Mori, K., 2001. XBP1 mRNA is induced by ATF6 and spliced by IRE1 in response to ER stress to produce a highly active transcription factor. Cell 107, 881–91.

SI Figures

A

Transcription Factor	Targets extrapolated from				
	JASPAR	Reactome	TRRUST	literature	Tot. No. targets genes
**XBP1**	30	33	14	91	168
**ATF6**	6	32	12	13	63
**ATF4-NRF2**	19	35	44	60	158

Biological process

B

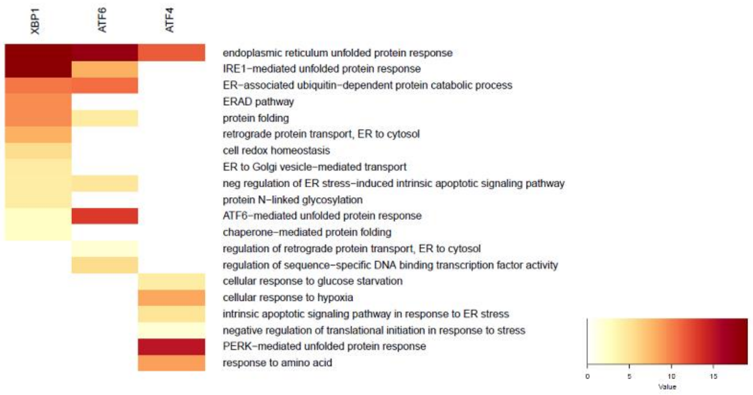

Cellular Component

C

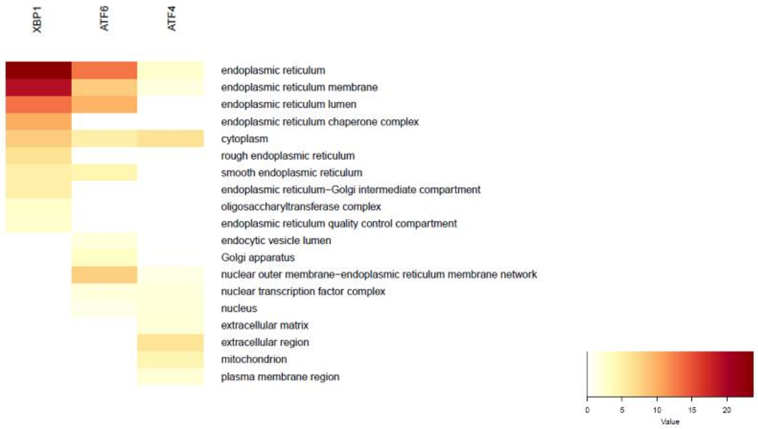

Molecular Function

D

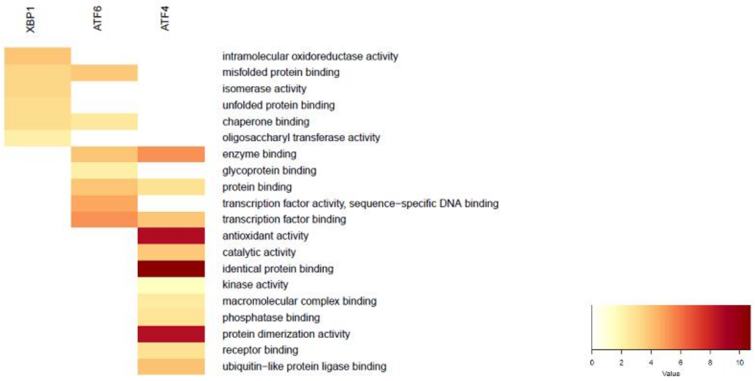

Figure S1. Gene Ontology (GO) analysis of obtained TFs target genes using DAVID and BioInfoMiner software. Target genes were classified by biological process, cellular component and molecular function. Target genes were divided by transcription factors (XBP1, ATF6, ATF4-NFE2L2) and a colour scale was used to determine the significance of the cluster enrichment. p-values of 10^{-20} was used as a maximum value for the colour scheme. A modified Fisher's exact P-value (EASE) was used to determine the gene-enrichment analysis and Benjamini-Hochberg multiple test correction was applied to calculated the False Discovery Rate (FDR). p-value significance:*P<0.01,**0.0001<P<0.001, ***0.00001<P<0.0001, ****P<0.0001.

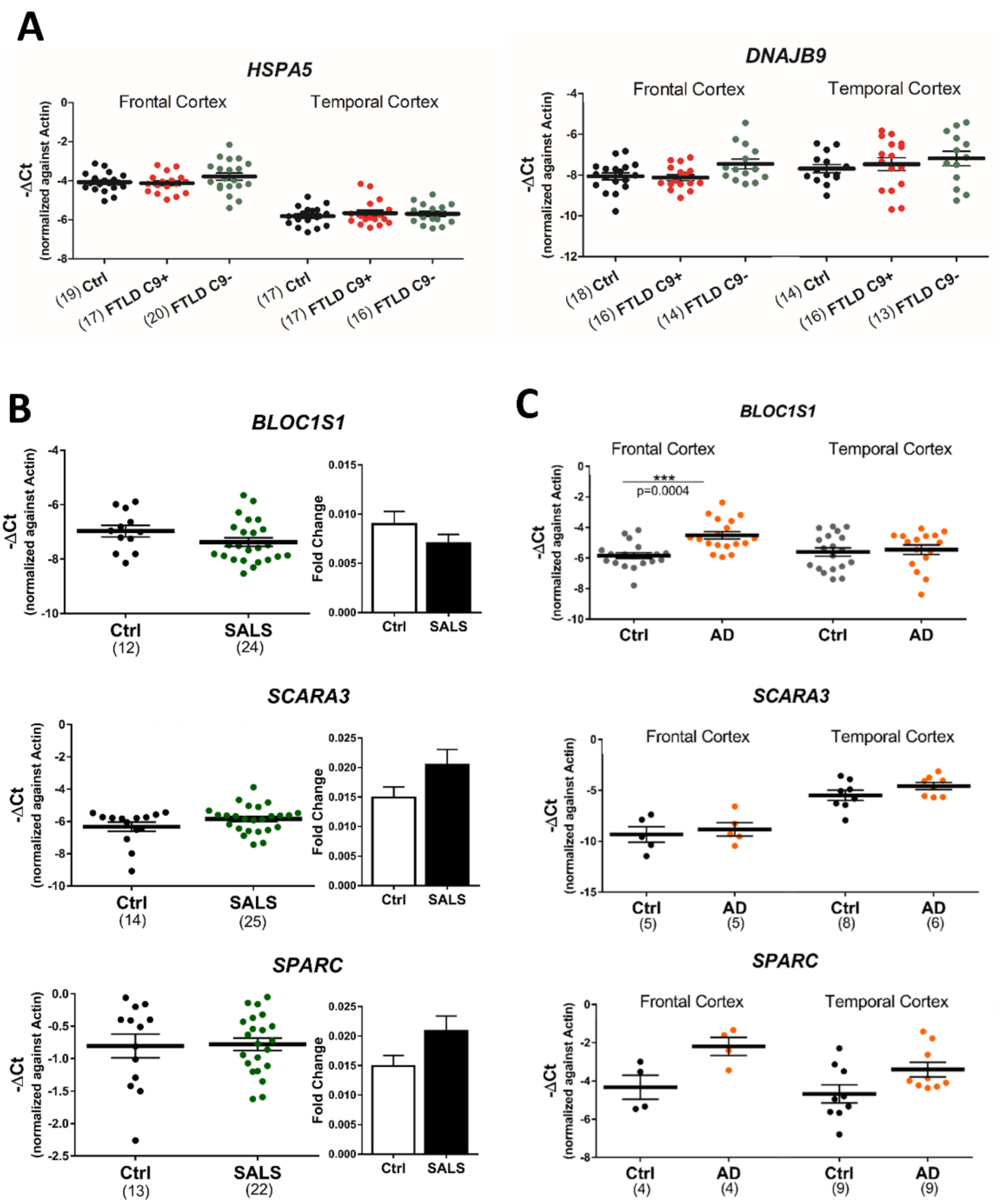

Figure S2. Gene expression analysis of XBP1 target genes tested in frontal and temporal cortex of brain derived from healthy individuals (Ctrl, grey) and patients affected by frontotemporal lobar dementia with (FTLD C9+, red) and without (FTLD C9-, green) C9orf72 repeat expansions. Means and SEMs were used to represent the data. The dots represent individual samples and the numbers under the graphs represent the number of samples analysed. Two-way ANOVA, followed by Tukey's multiple comparison tests or Kruskal-Wallis followed by Dunns multiple comparison tests were used depending on the data distribution (normal or non-parametric distribution, respectively). (B-C) Gene expression of a representative group of RIDD target genes in ALS and AD cases. Means and SEMs were used to represent the data. The dots represent individual samples and the numbers under the graphs represent the number of samples analysed. Unpaired *t*-test or Mann-Whitney U-test were used depending on the data distribution (normal or non-parametric distribution, respectively). *p<0.05; **p<0.01, ***p<0.001. FTLD, frontotemporal lobar dementia; Ctrl, control. ALS, amyotrophic lateral sclerosis; AD, Alzheimer's disease;

A

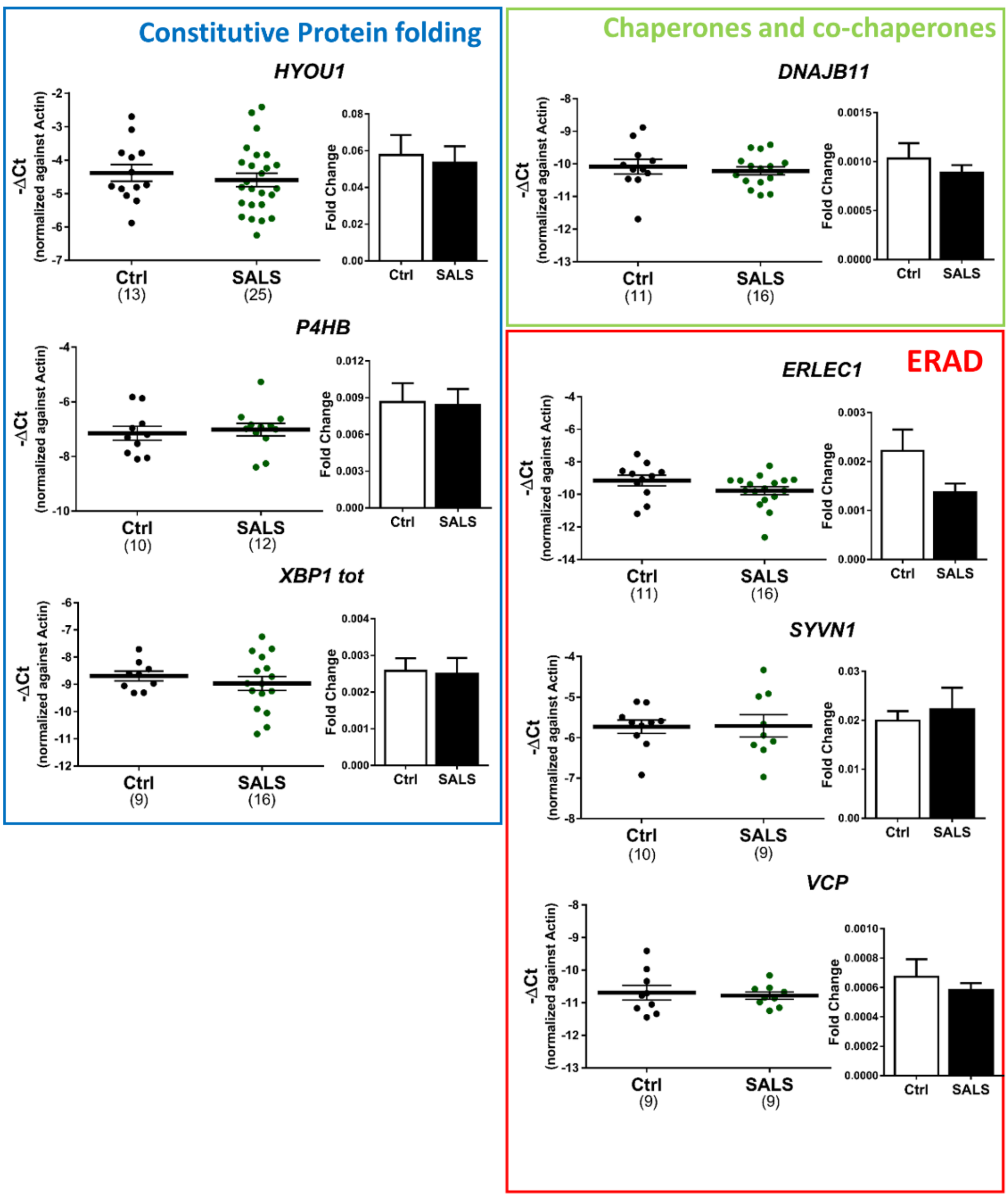

B

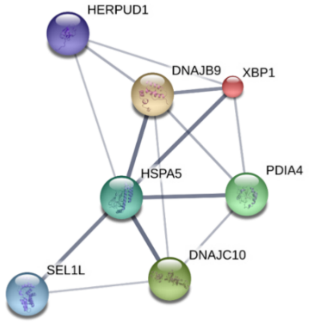

C

Biological Process (BP)

Pathway ID	*Pathway description (BP)*	*Count in gene set*	*FDR*
GO:0036503	ERAD pathway	5	5.75e-08
GO:0006511	Ubiquitin-dependent protein catabolic process	6	1.84e-06
GO:0044257	Cellular protein catabolic process	6	2.37e-06
GO:0034976	Response to ER stress	5	3.13e-06
GO:0030433	ER-associated ubiquitin-dependent protein catabolic process	4	3.25e-06

Figure S3. Gene expression of XBP1 target genes tested in spinal cord derived from sporadic cases of ALS (SALS). (A) Gene expression of a representative group of XBP1 target genes in spinal cord post-mortem samples derived from healthy individuals (Ctrl, black) and cases of amyotrophic lateral sclerosis (SALS, dark green). Genes were grouped by their main function. Means and SEMs were used to represent the data. The dots represent individual samples and the numbers under the graphs represent the number of samples analysed. SALS, sporadic amyotrophic lateral sclerosis; Ctrl, control. Unpaired *t*-test or Mann-Whitney U-test were used depending on the data distribution (normal or non-parametric distribution, respectively); *p<0.05; **p<0.01, ***p<0.001. (B) Protein-protein interaction network generated by STRING 10.5 (*Search Tool for the Retrieval of Interacting Genes/Proteins)* database. The connecting lines indicate functional relationships and direct protein-protein interactions. Line thickness indicates the strength of data support. Different colours reflects different proteins. (C) GO enrichment analysis was performed and first 5 terms of biological process (BP) are shown in the table. Count in gene set indicates the number of genes present in each category. Benjamini-Hochberg test was used to calculate False Discovery Rate (FDR).

A

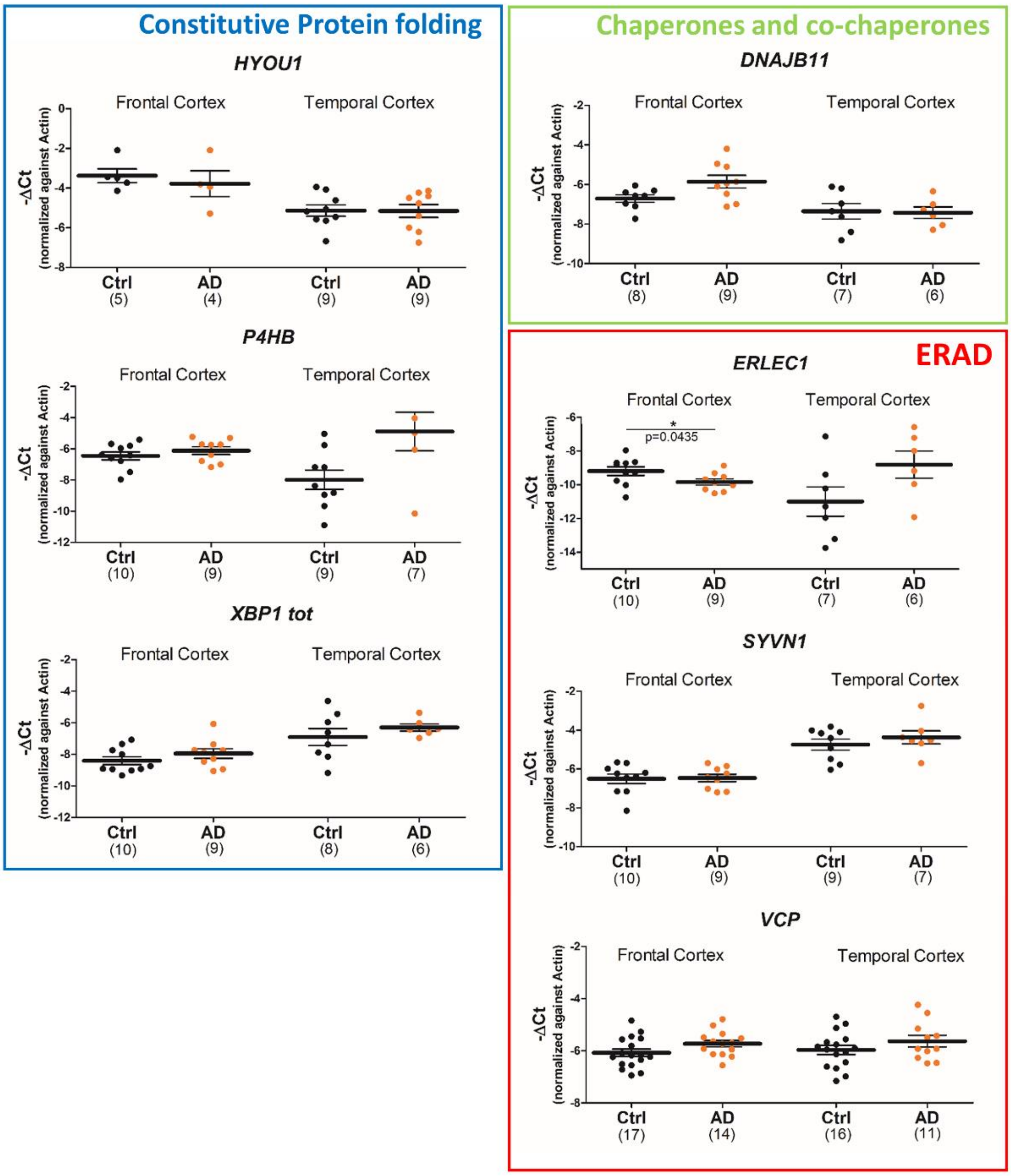

B

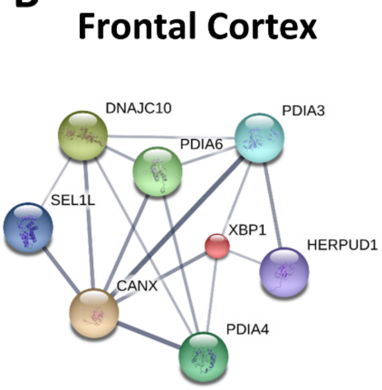

C

Biological Process (BP)

Pathway ID	*Pathway description (BP)*	*Count in gene set*	*FDR*
GO:0034975	Protein folding in ER	3	2.44e-05
GO:0034976	Response to ER stress	5	2.44e-05
GO:0045454	Cell redox homeostasis	4	2.44e-05
GO:0019725	Cellular homeostasis	6	5.93e-05
GO:0006457	Protein folding	4	0.000159

D

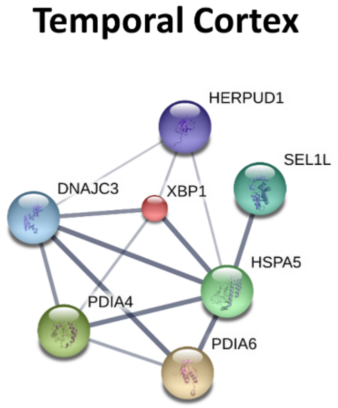

E

Biological Process (BP)

Pathway ID	*Pathway description (BP)*	*Count in gene set*	*FDR*
GO:0030968	ER unfolded protein response	5	5.61e-07
GO:0034620	Cellular response to unfolded protein	5	5.61e-07
GO:0034976	Response to ER stress	5	3.65e-06
GO:0036498	IRE1-mediated unfolded protein response	4	3.98e-06
GO:0051603	Proteolysis involved in cellular protein catabolic process	5	0.000169

Figure S4. (A) Gene expression analysis of XBP1 target genes tested in frontal and temporal cortex of brain derived from Alzheimer ‘s disease (AD) cases. Gene expression of a representative group of XBP1 target genes in frontal and temporal cortex post-mortem samples derived from healthy individuals (Ctrl, grey) and Alzheimer’s Disease cases (AD, orange). Genes were grouped by their main function. Means and SEMs were used to represent the data. The dots represent the individual samples and the numbers under the graphs represent the number of samples analysed. AD, Alzheimer’s disease; Ctrl, control. Unpaired *t*-test or Mann-Whitney U-test were used depending on the data distribution (normal or non-parametric distribution, respectively). *p<0.05; **p<0.01, ***p<0.001. (B-E) Protein-protein interaction network generated by STRING 10.5 (*Search Tool for the Retrieval of Interacting Genes/Proteins)* database on genes upregulated in (B) frontal and (D) temporal cortex. The connecting lines indicate functional relationships and direct protein-protein interactions. Line thickness indicates the strength of data support. Different colours reflects different proteins. GO enrichment analysis was performed and first 5 terms of biological process are shown in the table for (C) frontal and (E) temporal cortex. Count in gene set indicates the number of genes present in each category; Benjamini-Hochberg test was used to calculate False Discovery Rate (FDR).

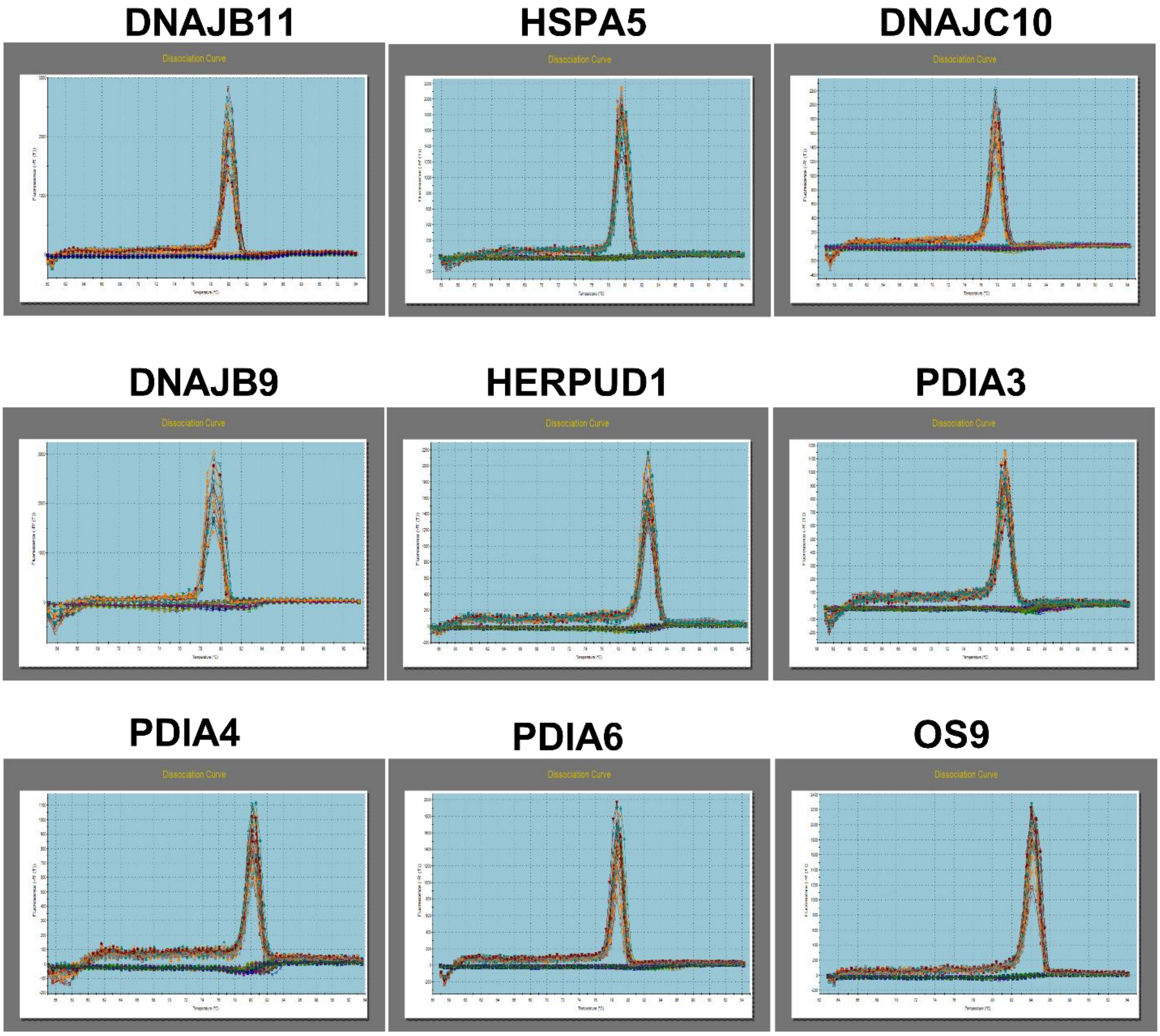

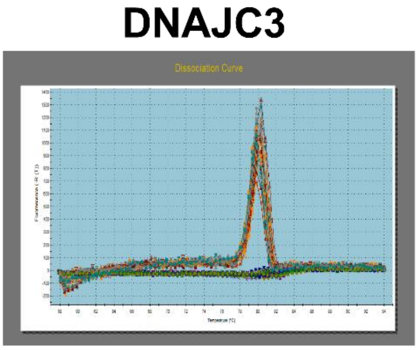

Figure S5. Dissociation curves for a representative group of genes tested in spinal cord derived from SALS cases and healthy individuals. Single product specific melting curves were obtained by melting curve analysis performed with Agilent Mx3000P QPCR System and analysed with MxPro program.

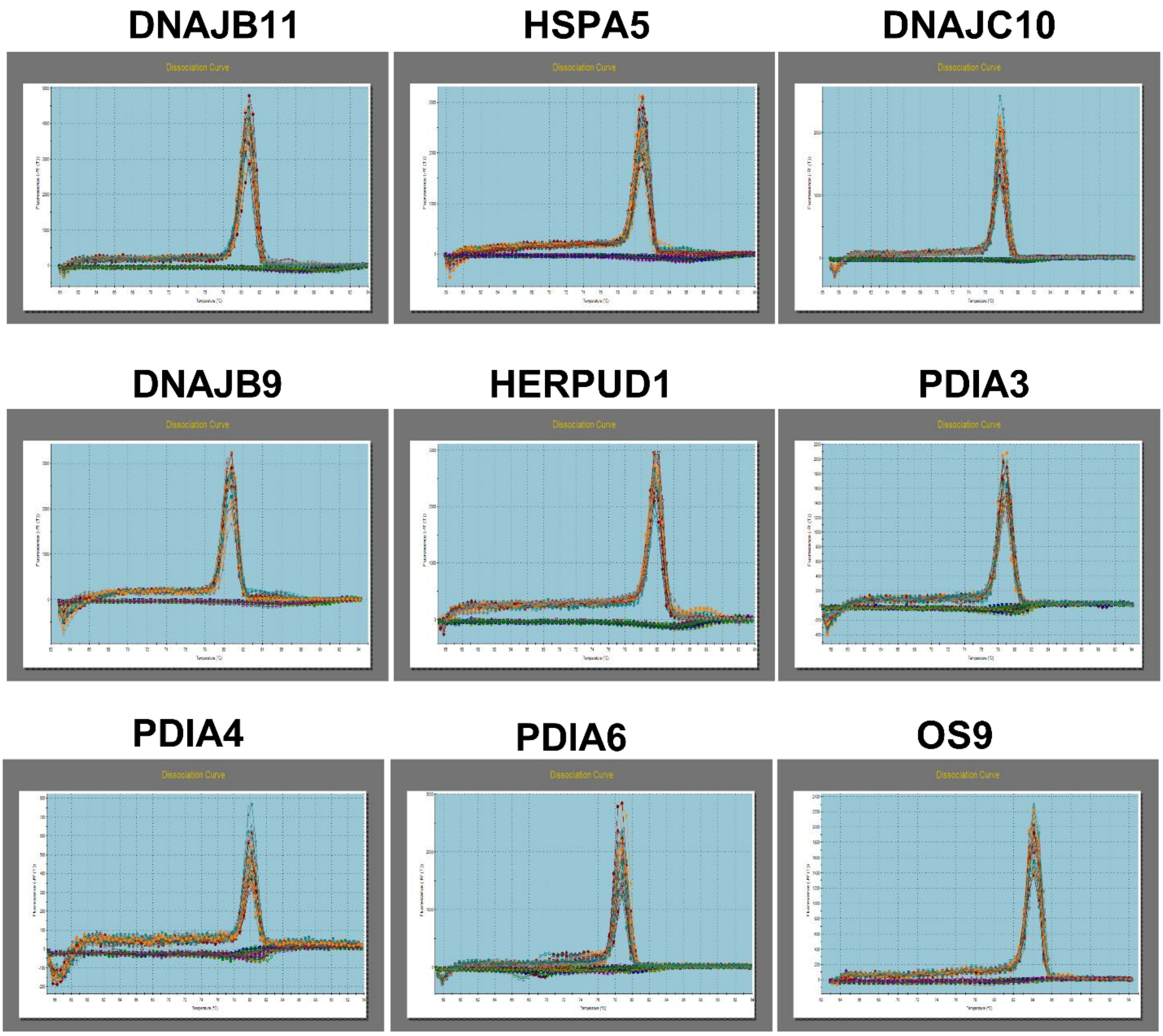

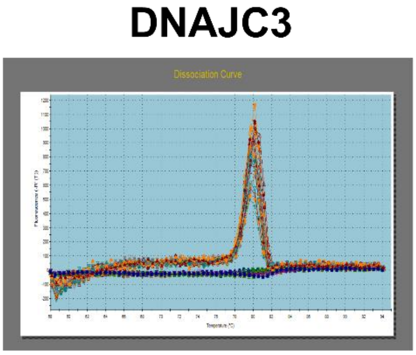

Figure S6. Dissociation curves for a representative group of genes tested in temporal cortex derived from AD cases and healthy individuals. Single product specific melting curves were obtained by melting curve analysis performed with Agilent Mx3000P QPCR System and analysed with MxPro program.

SI Tables

Table S1. Clinical details of patient samples, including gender, age at death, and post-mortem delay.

Group	n	Age at death (years) median (range)	Post-mortem delay (hours) median (range)
Controls	20 *male = 12* *female = 8*	68 (55 – 99)	46.5 (12 – 78)
Alzheimer's	20 *male = 9* *female = 11*	80 (53 – 89)	33.5 (3 – 73)
FTLD C9+	19 *male = 9* *female = 10*	62 (53 – 86)	22.5 (3 – 94)
FTLD C9-	20 *male = 11* *female = 9*	75 (61 - 92)	36.5 (5 – 72)

Group	n	Age at death (years) median (range)	Post-mortem delay (hours) median (range)
Controls	17 *male = 12* *female = 5*	66 (20 – 91)	11 (3 – 19)
SALS	32 *male = 21* *female = 11*	68 (24 – 85)	14 (5 – 25)

Table S2. List of Primers

Gene	Primers		Tm°
	**FORWARD**	**REVERSE**	
ACTIN B	CTGGAACGGTGAAGGTGACA	AAGGGACTTCCTGTAACAATGCA	57
BLOC1S1	GCACTCAAGGAAATTGGG	CCCTTTGTAGACATATTCCAG	56
CANX	GCCTCCGCCTCTCTCTTTAC	CCATGATCTCTAGCCTCCCG	62
DNAJB11	GGGAGCCTGGAGATTTACGG	CTTGTGACCATCCAAGTGAGT	60
DNAJB9	TTTCCAGACACGCCAGGATG	GTCCTGCAGTGCTTGCTAGA	63
DNAJC10	CAGTGAAATATCATGGAGACAG	ATTTCCTGTCCAAAGTTCTG	57
DNAJC3	TCGGGAATGTCTTAAACTTGACCA	TCAGCAATGCTTGGCTCTGT	58
ERLEC1	GCTCACTGTTGGGACAACCCACA	CCATGTCCCGACAACCACAGAGG	62
HERPUD1	GTTGGGTGGTTTCCATTTAG	CTTCCTGTAAGTTATTGTTGGG	55
HSPA5	TCAAGTTCTTGCCGTTCAAGG	AAATAAGCCTCAGCGGTTTCTT	58
HYOU1	GAAGATGCAGAGCCCATTTC	TCTGCTCCAGGACCTCCTAA	58
OS9	CAGCGTGAAAGGGAGGAGGAAA	GTGGTATTGCTGGATGTGGCGT	59
P4HB	TGTCATCGAGTTCACCGAGC	GTGGTCGCTGTCGATGAAGA	65
PDIA3	TCTGAAGAGATACCTGAAGTC	CAAAATTCTCTGCTACCACTAC	57
PDIA4	CAAGAGGTTTGATGTCTCTG	TTCCATATTTTTCTCGTGGG	55
PDIA6	GTGCAGTTGATGCAGATAAG	ACCTTGGTAATCTTCTGGTC	57
SEL1L	AGCCTACATGATGCAAACTGTG	AAGAGGCTAAGTGGTGAACAGG	57
SIL1	GCTGATCAACAAGTTCAAT	ATTCAAGATCAAAGAGCGC	57
SYVN1	GACAGTTCAAGAAAGCTGTG	TATGGAAAATGTGGTTGCAG	57
SCARA3	CGCTGCCAGAAGAACCTATC	AACCAGAGAGGCCAACACAG	57
SPARC	GGCCTGGATCTTCTTTCTCC	CCACCACCTCTGTCTCATCA	58
VCP	AAACCGTGGTAGAGGTGCCA	CTTGGAAGGTGTCATGCCAA	62
XBP1 tot	GCTCAGACTGCCAGAGATCG	TCTTCAGCAACCAGGGCATC	63
XBP1s	AGAGTCTGATATCCTGTTGG	AGTTCATTAATGGCTTCCAG	57

Table S3

XBP1 target genes (source:JASPAR)	XBP1 target genes (source:Reactome)	XBP1 target genes (source:TRRUST)	XBP1 target genes (source:Literature)		XBP1 target genes (total list)	
CANX	ACADVL	CCL2	ALG12	VCP	ACADVL	MANF
CHKB	ADD1	CXCL8	ALG2	VEPH1	ADD1	MYDGF
DNAJB11	ARFGAP1	ESR1	ALOX12B	YPEL3	ALG12	MYEOV2
DNAJB9	ATP6V0D1	FOXO1	ATG13	ZNF17	ALG2	NAT6
DNAJC10	CTDSP2	GAD1	ATXN1L	ZNF721	ALOX12B	NLRP4
DNAJC3	CUL7	HSPA1A	BFAR	ZPBP2	ARFGAP1	NOL10
EDEM1	CXXC1	IL6	C16orf78		ATG13	NOS2
EDEM2	DCTN1	NOS2	C6orf118		ATP6V0D1	NPPB
ERLEC1	DDX11	NPPB	C6orf201		ATXN1L	NUCB2
ERN1	EXTL3	PRNP	CAV2		BFAR	NUDT9
HERPUD1	FKBP14	PTGER4	CHST7		C16orf78	P4HB
HSPA5	GFPT1	SOD2	CKMT2		C6orf118	PCDHGB1
HYOU1	GOSR2	VEGFA	COPG1		C6orf201	PDIA3
IGF1	GSK3A	ZHX2	CREB3L2		CANX	PDIA4
MANF	HDGF		CRTAP		CAV2	PDIA5
NUCB2	KDELR3		DAD1		CCL2	PDIA6
P4HB	KLHDC3		DDX50		CHKB	PECR
PDIA3	LMNA		DERL1		CHST7	PHKA1P1
PDIA4	MYDGF		EIF2A		CKMT2	PLA2G4B
PDIA5	PLA2G4B		EML1		COPG1	PPIB
PDIA6	PPP2R5B		ERP29		CREB3L2	PPP1R3G
SEC61A1	PREB		ERP44		CRTAP	PPP2R5B
SEC61B	SEC31A		FAM135A		CTDSP2	PREB
SERP1	SHC1		FAM83F		CUL7	PRM3
SPG7	SSR1		FICD		CXCL8	PRNP
SSR4	SULT1A3		FKBP2		CXXC1	PTGER4
STT3A	TATDN2		FOXO4		DAD1	REEP5
SYVN1	TLN1		FRG2		DCTN1	RPN1
WFS1	TPP1		FRG2B		DDX11	RPN2
XBP1	TSPYL2		GAD2		DDX50	RPS16P6
	WIPI1		GADD45B		DERL1	SEC31A
	YIF1A		GIT2		DNAJB11	SEC61A1
	ZBTB17		GJB2		DNAJB9	SEC61B
			GLRA2		DNAJC10	SEC63
			GNAQ		DNAJC3	SERBP1
			HADH		EDEM1	SERP1
			HSP90B1		EDEM2	SERPIND1
			HSPA13		EIF2A	SESN2
			HYAL3		EML1	SHC1
			IMPG1		ERLEC1	SHE
			KBTBD2		ERN1	SLC33A1
			L3MBTL2		ERP29	SLC38A5
			LOC100131680		ERP44	SMPD1
			LPIN3		ESR1	SNORA71B
			LRFN4		EXTL3	SOD2
			LRRC55		FAM135A	SPG7
			MYEOV2		FAM83F	SPTBN5
			NAT6		FICD	SRPRA
			NLRP4		FKBP14	SRPRB
			NOL10		FKBP2	SSR1
			NUDT9		FOXO1	SSR3
			PCDHGB1		FOXO4	SSR4
			PECR		FRG2	STT3A
			PHKA1P1		FRG2B	STX5
			PPIB		GAD1	SULT1A3
			PPP1R3G		GAD2	SYVN1
			PRM3		GADD45B	TATDN2
			REEP5		GFPT1	TGS1
			RPN1		GIT2	TIMM44
			RPN2		GJB2	TLN1
			RPS16P6		GLRA2	TMCO1
			SEC63		GNAQ	TMEM168
			SERBP1		GOSR2	TMEM175
			SERPIND1		GSK3A	TMEM87A
			SESN2		HADH	TPP1
			SHE		HDGF	TSPYL2
			SLC33A1		HERPUD1	TXNDC11
			SLC38A5		HSP90B1	UBE2E2
			SMPD1		HSPA13	USP34
			SNORA71B		HSPA1A	USP4
			SPTBN5		HSPA5	VCP
			SRPRA		HYAL3	VEGFA
			SRPRB		Hyou1	VEPH1
			SSR3		IGF1	WFS1
			STX5		IL6	WIPI1
			TGS1		IMPG1	XBP1
			TIMM44		KBTBD2	YIF1A
			TMCO1		KDELR3	YPEL3
			TMEM168		KLHDC3	ZBTB17
			TMEM175		L3MBTL2	ZHX2
			TMEM87A		LMNA	ZNF17
			TXNDC11		LOC100131680	ZNF721
			UBE2E2		LPIN3	ZPBP2
			USP34		LRFN4	
			USP4		LRRC55	

**ATF6 target genes (source:JASPAR)**	**ATF6 target genes (source:Reactome)**	**ATF6 target genes (source:TRRUST)**	**ATF6 target genes (source:Literature)**	**ATF6 target genes (total list)**
CALR	APP	ATP2A2	CRELD2	APP
EDEM1	ATF6B	BGLAP	DERL3	ATF6B
ERO1L	BPGM	CEBPB	DNAJB11	ATP2A2
HERPUD1	CREB1	DNAJC3	ERO1LB	BGLAP
OS9	CREB3L3	HSPA5	GALNT3	BPGM
PDIA3	CREBZF	HYOU1	HSP90B1	CALR
	CRTC2	MAFA	MANF	CEBPB
	DDC	NR0B2	ORMDL2	CREB1
	FBXO6	NUCB1	PDIA4	CREB3L3
	GTF2I	PDX1	PDIA6	CREBZF
	HNF4A	RTN3	SDF2L1	CRELD2
	MAPK1	WBP1	SEL1L	CRTC2
	MAPK11		WIPI1	DDC
	MAPK14			DERL3
	MAPK3			DNAJB11
	MAX			DNAJC3
	MBTPS1			EDEM1
	MBTPS2			ERO1L
	MYC			ERO1LB
	NFYA			FBXO6
	NFYC			GALNT3
	NNMT			GTF2I
	SAR1A			HERPUD1
	SREBF2			HNF4A
	SRF			HSP90B1
	SUMO2			HSPA5
	TAF1			HYOU1
	TNFRSF1A			MAFA
	UBC			MANF
	WFS1			MAPK1
	XBP1			MAPK11
	YY1			MAPK14
				MAPK3
				MAX
				MBTPS1
				MBTPS2
				MYC
				NFYA
				NFYC
				NNMT
				NR0B2
				NUCB1
				ORMDL2
				OS9
				PDIA3
				PDIA4
				PDIA6
				PDX1
				RTN3
				SAR1A
				SDF2L1
				SEL1L
				SREBF2
				SRF
				SUMO2
				TAF1
				TNFRSF1A
				UBC
				WBP1
				WFS1
				WIPI1
				XBP1
				YY1

**ATF4 target genes (source:JASPAR)**	**ATF4 target genes (source:Reactome)**	**ATF4 target genes (source:TRRUST)**	**ATF4 target genes (source:Literature)**	**ATF4 target genes (total list)**	
APOE	BNIP3L	ASNS	AQP44	APOE	IL8
ATF5	CCDC130	ATF3	ATF4	AQP44	IRF7
ATG16L2	CHAC1	BGLAP	ATF6	ASNS	LCLAT1
CHOP	CLSTN2	CA9	BIRC2	ATF3	LOXL1
COX6A2	DKFZP434K028	CCL2	CARS	ATF4	LTBP1
CTH	DKFZP686K1684	CEBPB	CASP8	ATF5	MAP1LC3A
EGR1	DST	DDIT3	CAV1	ATF6	MAP1LC3B
EIF4EBP1	EIF1	DDIT4	EGFR	ATG16L2	MAPK1
HERPUD1	ETNK1	DDR2	EIF2A	BGLAP	MAPK3
HES5	FOXD3	DISC1	EIF2AK2	BIRC2	MCL1
MTHFD2	FOXP2	FGF19	EIF2AK3	BNIP3L	MED29
OVGP1	HHEX	FGF2	EIF2S1	CA9	MGAT3
SERPINF1	HOXD11	FGF21	ERP29	CARS	MMP2
SLC3A2	LCLAT1	HSPA5	FAS	CASP8	MTHFD2
SLC6A9	LTBP1	IGFBP1	FGFR3	CAV1	MTOR
SLC7A3	MAP1LC3A	IL6	HIF1A	CCDC130	NARS
	MED29	IRF7	HOXB7	CCL2	NDC80
	OBSCN	MAP1LC3B	IL6R	CEBPB	NFKB1
	PHACTR3	MCL1	IL8	CHAC1	NOS3
	RAB7A	NDC80	LOXL1	CHOP	NUPR1
	RCN1	NUPR1	MAPK1	CLSTN2	OBSCN
	SLC25A36	PLAU	MAPK3	COX6A2	OVGP1
	TRIB1	POLR2C	MGAT3	CTH	PARP16
	WNT10A	S100P	MMP2	DDIT3	PHACTR3
	ZC3H12C	SIGMAR1	MTOR	DDIT4	PKD2
		SIRT1	NARS	DDR2	PLAU
		SIRT2	NFKB1	DISC1	POLR2C
		TNFRSF10B	NOS3	DKFZP434K028	PSAT1
		TRIB3	PARP16	DKFZP686K1684	RAB7A
		VEGFA	PKD2	DST	RCN1
			PSAT1	EGFR	RHOA
			RHOA	EGR1	S100P
			SERPINI1	EIF1	SERPINF1
			SHMT2	EIF2A	SERPINI1
			SLC1A4	EIF2AK2	SHMT2
			SLC25A5	EIF2AK3	SIGMAR1
			SOX21	EIF2S1	SIRT1
			SPON2	EIF4EBP1	SIRT2
			STAT3	ERP29	SLC1A4
			TGFBI	ETNK1	SLC25A36
			TRB3	FAS	SLC25A5
				FGF19	SLC3A2
				FGF2	SLC6A9
				FGF21	SLC7A3
				FGFR3	SOX21
				FOXD3	SPON2
				FOXP2	STAT3
				HERPUD1	TGFBI
				HES5	TNFRSF10B
				HHEX	TRB3
				HIF1A	TRIB1
				HOXB7	TRIB3
				HOXD11	VEGFA
				HSPA5	WNT10A
				IGFBP1	ZC3H12C
				IL6	
				IL6R	

NFE2L2 target genes (source:JASPAR)	NFE2L2 target genes (source:Reactome)	NFE2L2 target genes (source:TRRUST)	NFE2L2 target genes (source:Literature)	NFE2L2 target genes (total list)	ATF4-NFE2L2 target genes (total list)	
GSTA1	BTG2	BRCA1	FTH	BRCA1	APOE	LTBP1
NQO1	CREBBP	CAT	FTL	BTG2	AQP44	MAP1LC3A
GCLC	KEAP1	CFTR	G6PD	CAT	ASNS	MAP1LC3B
	KMT2D	CXCL8	GCLM	CFTR	ATF3	MAPK1
	NFE2L2	GSS	GPX2	CREBBP	ATF4	MAPK3
	PIK3CA	HBB	GSR	CXCL8	ATF5	MCL1
	PTEN	HMOX1	GSTA3	FTH	ATF6	ME1
	RB1	KRT16	GSTA5	FTL	ATG16L2	MED29
	TP53	MGST1	GSTM1	G6PD	BGLAP	MGAT3
	UCP1	MTHFR	GSTM3	GCLC	BIRC2	MGST1
		SDHB	GSTP1	GCLM	BNIP3L	MMP2
		SOD1	HMOX	GPX2	BRCA1	MTHFD2
		SOD2	IDH1	GSR	BTG2	MTHFR
		UGT2B7	ME1	GSS	CA9	MTOR
			PGD	GSTA1	CARS	NARS
			PRDX1	GSTA3	CASP8	NDC80
			TXN1	GSTA5	CAT	NFE2L2
			TXNRD1	GSTM1	CAV1	NFKB1
			XCT	GSTM3	CCDC130	NOS3
				GSTP1	CCL2	NQO1
				HBB	CEBPB	NUPR1
				HMOX	CFTR	OBSCN
				HMOX1	CHAC1	OVGP1
				IDH1	CHOP	PARP16
				KEAP1	CLSTN2	PGD
				KMT2D	COX6A2	PHACTR3
				KRT16	CREBBP	PIK3CA
				ME1	CTH	PKD2
				MGST1	CXCL8	PLAU
				MTHFR	DDIT3	POLR2C
				NFE2L2	DDIT4	PRDX1
				NQO1	DDR2	PSAT1
				PGD	DISC1	PTEN
				PIK3CA	DKFZP434K028	RAB7A
				PRDX1	DKFZP686K1684	RB1
				PTEN	DST	RCN1
				RB1	EGFR	RHOA
				SDHB	EGR1	S100P
				SOD1	EIF1	SDHB
				SOD2	EIF2A	SERPINF1
				TP53	EIF2AK2	SERPINI1
				TXN1	EIF2AK3	SHMT2
				TXNRD1	EIF2S1	SIGMAR1
				UCP1	EIF4EBP1	SIRT1
				UGT2B7	ERP29	SIRT2
				XCT	ETNK1	SLC1A4
				XCT	FAS	SLC25A36
					FGF19	SLC25A5
					FGF2	SLC3A2
					FGF21	SLC6A9
					FGFR3	SLC7A3
					FOXD3	SOD1
					FOXP2	SOD2
					FTH	SOX21
					FTL	SPON2
					G6PD	STAT3
					GCLC	TGFBI
					GCLM	TNFRSF10B
					GPX2	TP53
					GSR	TRB3
					GSS	TRIB1
					GSTA1	TRIB3
					GSTA3	TXN1
					GSTA5	TXNRD1
					GSTM1	UCP1
					GSTM3	UGT2B7
					GSTP1	VEGFA
					HBB	WNT10A
					HERPUD1	XCT
					HES5	ZC3H12C
					HHEX	
					HIF1A	
					HMOX	
					HMOX1	
					HOXB7	
					HOXD11	
					HSPA5	
					IDH1	
					IGFBP1	
					IL6	
					IL6R	
					IL8	
					IRF7	
					KEAP1	
					KMT2D	
					KRT16	
					LCLAT1	
					LOXL1	

**XBP1 target genes (total list)**		**Merge**	**ATF6 target genes (total list)**
ACADVL	NAT6	DNAJB11	APP
ADD1	NLRP4	DNAJC3	ATF6B
ALG12	NOL10	EDEM1	ATP2A2
ALG2	NOS2	HERPUD1	BGLAP
ALOX12B	NPPB	HSP90B1	BPGM
ARFGAP1	NUCB2	HSPA5	CALR
ATG13	NUDT9	HYOU1	CEBPB
ATP6V0D1	P4HB	MANF	CREB1
ATXN1L	PCDHGB1	PDIA3	CREB3L3
BFAR	PDIA3	PDIA4	CREBZF
C16orf78	PDIA4	PDIA6	CRELD2
C6orf118	PDIA5	WFS1	CRTC2
C6orf201	PDIA6	WIPI1	DDC
CANX	PECR	XBP1	DERL3
CAV2	PHKA1P1		DNAJB11
CCL2	PLA2G4B		DNAJC3
CHKB	PPIB		EDEM1
CHST7	PPP1R3G		ERO1L
CKMT2	PPP2R5B		ERO1LB
COPG1	PREB		FBXO6
CREB3L2	PRM3		GALNT3
CRTAP	PRNP		GTF2I
CTDSP2	PTGER4		HERPUD1
CUL7	REEP5		HNF4A
CXCL8	RPN1		HSP90B1
CXXC1	RPN2		HSPA5
DAD1	RPS16P6		HYOU1
DCTN1	SEC31A		MAFA
DDX11	SEC61A1		MANF
DDX50	SEC61B		MAPK1
DERL1	SEC63		MAPK11
DNAJB11	SERBP1		MAPK14
DNAJB9	SERP1		MAPK3
DNAJC10	SERPIND1		MAX
DNAJC3	SESN2		MBTPS1
EDEM1	SHC1		MBTPS2
EDEM2	SHE		MYC
EIF2A	SLC33A1		NFYA
EML1	SLC38A5		NFYC
ERLEC1	SMPD1		NNMT
ERN1	SNORA71B		NR0B2
ERP29	SOD2		NUCB1
ERP44	SPG7		ORMDL2
ESR1	SPTBN5		OS9
EXTL3	SRPRA		PDIA3
FAM135A	SRPRB		PDIA4
FAM83F	SSR1		PDIA6
FICD	SSR3		PDX1
FKBP14	SSR4		RTN3
FKBP2	STT3A		SAR1A
FOXO1	STX5		SDF2L1
FOXO4	SULT1A3		SEL1L
FRG2	SYVN1		SREBF2
FRG2B	TATDN2		SRF
GAD1	TGS1		SUMO2
GAD2	TIMM44		TAF1
GADD45B	TLN1		TNFRSF1A
GFPT1	TMCO1		UBC
GIT2	TMEM168		WBP1
GJB2	TMEM175		WFS1
GLRA2	TMEM87A		WIPI1
GNAQ	TPP1		XBP1
GOSR2	TSPYL2		YY1
GSK3A	TXNDC11		
HADH	UBE2E2		
HDGF	USP34		
HERPUD1	USP4		
HSP90B1	VCP		
HSPA13	VEGFA		
HSPA1A	VEPH1		
HSPA5	WFS1		
HYAL3	WIPI1		
Hyou1	XBP1		
IGF1	YIF1A		
IL6	YPEL3		
IMPG1	ZBTB17		
KBTBD2	ZHX2		
KDELR3	ZNF17		
KLHDC3	ZNF721		
L3MBTL2	ZPBP2		
LMNA			
LOC100131680			
LPIN3			
LRFN4			
LRRC55			
MANF			
MYDGF			
MYEOV2			

**XBP1 target genes (total list)**		**Merge**	**ATF4-NFE2L2 target genes (total list)**	
ACADVL	NAT6	CCL2	APOE	LTBP1
ADD1	NLRP4	CXCL8	AQP44	MAP1LC3A
ALG12	NOL10	EIF2A	ASNS	MAP1LC3B
ALG2	NOS2	ERP29	ATF3	MAPK1
ALOX12B	NPPB	HERPUD1	ATF4	MAPK3
ARFGAP1	NUCB2	HSPA5	ATF5	MCL1
ATG13	NUDT9	IL6	ATF6	ME1
ATP6V0D1	P4HB	SOD2	ATG16L2	MED29
ATXN1L	PCDHGB1	VEGFA	BGLAP	MGAT3
BFAR	PDIA3		BIRC2	MGST1
C16orf78	PDIA4		BNIP3L	MMP2
C6orf118	PDIA5		BRCA1	MTHFD2
C6orf201	PDIA6		BTG2	MTHFR
CANX	PECR		CA9	MTOR
CAV2	PHKA1P1		CARS	NARS
CCL2	PLA2G4B		CASP8	NDC80
CHKB	PPIB		CAT	NFE2L2
CHST7	PPP1R3G		CAV1	NFKB1
CKMT2	PPP2R5B		CCDC130	NOS3
COPG1	PREB		CCL2	NQO1
CREB3L2	PRM3		CEBPB	NUPR1
CRTAP	PRNP		CFTR	OBSCN
CTDSP2	PTGER4		CHAC1	OVGP1
CUL7	REEP5		CHOP	PARP16
CXCL8	RPN1		CLSTN2	PGD
CXXC1	RPN2		COX6A2	PHACTR3
DAD1	RPS16P6		CREBBP	PIK3CA
DCTN1	SEC31A		CTH	PKD2
DDX11	SEC61A1		CXCL8	PLAU
DDX50	SEC61B		DDIT3	POLR2C
DERL1	SEC63		DDIT4	PRDX1
DNAJB11	SERBP1		DDR2	PSAT1
DNAJB9	SERP1		DISC1	PTEN
DNAJC10	SERPIND1		DKFZP434K028	RAB7A
DNAJC3	SESN2		DKFZP686K1684	RB1
EDEM1	SHC1		DST	RCN1
EDEM2	SHE		EGFR	RHOA
EIF2A	SLC33A1		EGR1	S100P
EML1	SLC38A5		EIF1	SDHB
ERLEC1	SMPD1		EIF2A	SERPINF1
ERN1	SNORA71B		EIF2AK2	SERPINI1
ERP29	SOD2		EIF2AK3	SHMT2
ERP44	SPG7		EIF2S1	SIGMAR1
ESR1	SPTBN5		EIF4EBP1	SIRT1
EXTL3	SRPRA		ERP29	SIRT2
FAM135A	SRPRB		ETNK1	SLC1A4
FAM83F	SSR1		FAS	SLC25A36
FICD	SSR3		FGF19	SLC25A5
FKBP14	SSR4		FGF2	SLC3A2
FKBP2	STT3A		FGF21	SLC6A9
FOXO1	STX5		FGFR3	SLC7A3
FOXO4	SULT1A3		FOXD3	SOD1
FRG2	SYVN1		FOXP2	SOD2
FRG2B	TATDN2		FTH	SOX21
GAD1	TGS1		FTL	SPON2
GAD2	TIMM44		G6PD	STAT3
GADD45B	TLN1		GCLC	TGFBI
GFPT1	TMCO1		GCLM	TNFRSF10B
GIT2	TMEM168		GPX2	TP53
GJB2	TMEM175		GSR	TRB3
GLRA2	TMEM87A		GSS	TRIB1
GNAQ	TPP1		GSTA1	TRIB3
GOSR2	TSPYL2		GSTA3	TXN1
GSK3A	TXNDC11		GSTA5	TXNRD1
HADH	UBE2E2		GSTM1	UCP1
HDGF	USP34		GSTM3	UGT2B7
HERPUD1	USP4		GSTP1	VEGFA
HSP90B1	VCP		HBB	WNT10A
HSPA13	VEGFA		HERPUD1	XCT
HSPA1A	VEPH1		HES5	ZC3H12C
HSPA5	WFS1		HHEX	
HYAL3	WIPI1		HIF1A	
Hyou1	XBP1		HMOX	
IGF1	YIF1A		HMOX1	
IL6	YPEL3		HOXB7	
IMPG1	ZBTB17		HOXD11	
KBTBD2	ZHX2		HSPA5	
KDELR3	ZNF17		IDH1	
KLHDC3	ZNF721		IGFBP1	
L3MBTL2	ZPBP2		IL6	
LMNA			IL6R	
LOC100131680			IL8	
LPIN3			IRF7	
LRFN4			KEAP1	
LRRC55			KMT2D	
MANF			KRT16	
MYDGF			LCLAT1	
MYEOV2			LOXL1	

ATF6 target genes (total list)	Merge	ATF4-NFE2L2 target genes (total list)	
APP	BGLAP	APOE	LTBP1
ATF6B	CEBPB	AQP44	MAP1LC3A
ATP2A2	HERPUD1	ASNS	MAP1LC3B
BGLAP	HSPA5	ATF3	MAPK1
BPGM	MAPK1	ATF4	MAPK3
CALR	MAPK3	ATF5	MCL1
CEBPB		ATF6	ME1
CREB1		ATG16L2	MED29
CREB3L3		BGLAP	MGAT3
CREBZF		BIRC2	MGST1
CRELD2		BNIP3L	MMP2
CRTC2		BRCA1	MTHFD2
DDC		BTG2	MTHFR
DERL3		CA9	MTOR
DNAJB11		CARS	NARS
DNAJC3		CASP8	NDC80
EDEM1		CAT	NFE2L2
ERO1L		CAV1	NFKB1
ERO1LB		CCDC130	NOS3
FBXO6		CCL2	NQO1
GALNT3		CEBPB	NUPR1
GTF2I		CFTR	OBSCN
HERPUD1		CHAC1	OVGP1
HNF4A		CHOP	PARP16
HSP90B1		CLSTN2	PGD
HSPA5		COX6A2	PHACTR3
HYOU1		CREBBP	PIK3CA
MAFA		CTH	PKD2
MANF		CXCL8	PLAU
MAPK1		DDIT3	POLR2C
MAPK11		DDIT4	PRDX1
MAPK14		DDR2	PSAT1
MAPK3		DISC1	PTEN
MAX		DKFZP434K028	RAB7A
MBTPS1		DKFZP686K1684	RB1
MBTPS2		DST	RCN1
MYC		EGFR	RHOA
NFYA		EGR1	S100P
NFYC		EIF1	SDHB
NNMT		EIF2A	SERPINF1
NR0B2		EIF2AK2	SERPINI1
NUCB1		EIF2AK3	SHMT2
ORMDL2		EIF2S1	SIGMAR1
OS9		EIF4EBP1	SIRT1
PDIA3		ERP29	SIRT2
PDIA4		ETNK1	SLC1A4
PDIA6		FAS	SLC25A36
PDX1		FGF19	SLC25A5
RTN3		FGF2	SLC3A2
SAR1A		FGF21	SLC6A9
SDF2L1		FGFR3	SLC7A3
SEL1L		FOXD3	SOD1
SREBF2		FOXP2	SOD2
SRF		FTH	SOX21
SUMO2		FTL	SPON2
TAF1		G6PD	STAT3
TNFRSF1A		GCLC	TGFBI
UBC		GCLM	TNFRSF10B
WBP1		GPX2	TP53
WFS1		GSR	TRB3
WIPI1		GSS	TRIB1
XBP1		GSTA1	TRIB3
YY1		GSTA3	TXN1
		GSTA5	TXNRD1
		GSTM1	UCP1
		GSTM3	UGT2B7
		GSTP1	VEGFA
		HBB	WNT10A
		HERPUD1	XCT
		HES5	ZC3H12C
		HHEX	
		HIF1A	
		HMOX	
		HMOX1	
		HOXB7	
		HOXD11	
		HSPA5	
		IDH1	
		IGFBP1	
		IL6	
		IL6R	
		IL8	
		IRF7	
		KEAP1	
		KMT2D	
		KRT16	
		LCLAT1	
		LOXL1	

Merge XBP1-ATF6	Merge XBP1-ATF4	Merge ATF6-ATF4	Merge
DNAJB11	CCL2	BGLAP	HERPUD1
DNAJC3	CXCL8	CEBPB	HSPA5
EDEM1	EIF2A	HERPUD1	
HERPUD1	ERP29	HSPA5	
HSP90B1	HERPUD1	MAPK1	
HSPA5	HSPA5	MAPK3	
HYOU1	IL6		
MANF	SOD2		
PDIA3	VEGFA		
PDIA4			
PDIA6			
WFS1			
WIPI1			
XBP1			

Table S4. Selected XBP1 and ATF6 target genes

Name	RefSeq	Alias	Localization	ER response elements	Position realative to the TSS	Function (UniProt)	XBP1 targets (Ref)	ATF6 targets (Ref)
ARF4	NM_001660.3	ARF2	Golgi	ACGT core<br>UPRE	- 239<br>-425	Involved in protein trafficking; may modulate vesicle budding and uncoating within the Golgi apparatus.	1	
CANX	NM_001024649.1	CNX; P90; IP90	ER	ERSE	-109	It seems to play a major role in the quality control apparatus of the ER by the retention of incorrectly folded proteins. Ca2+ binfing protein.	1	
CHKB	NM_005198.4	CK; EK; CKB	Cytosol	ACGT core<br>CRE-like	+ 4394<br>+1306	Has a key role in phospholipid biosynthesis.	1	
DAD1	NM_001344.3	OST2	ER membrane	ACGT core	- 245	Loss of the DAD1 protein triggers apoptosis	2	
DERL1	NM_024295.5	DER1; DER-1	ER membrane	ERSE<br>ACGT core	-141<br>-138	Functional component of endoplasmic reticulum-associated degradation (ERAD) for misfolded lumenal proteins.	3,45	
DNAJB11	NM_016306.5	HEDJ; ERdj3;	ER, secreted	3X ERSE<br>ACGT core	-357<br>-2 238	Serves as a co-chaperone for HSPA5. Binds directly to both unfolded proteins	5–8	9
DNAJB9	NM_012328.2	ERdj4, MDG-1, MDG1,	ER	CRE-like<br>ACGT core	- 1955<br>-59	Involved in endoplasmic reticulum-associated degradation (ERAD) of misfolded proteins.	1,6,10,11	
DNAJC10	NM_001271581.1	ERdj5, PDIA19	ER	UPRE	-132	Endoplasmic reticulum disulfide reductase involved both in the correct folding of proteins and degradation of misfolded proteins.	12	
DNAJC3	NM_006260.4	P58IPK; ERdj6;	ER	ERSE	-233	Involved in the unfolded protein response (UPR) during endoplasmic reticulum (ER) stress.	6,7,10,11	13–15
EDEM1	NM_014674.2	__	ER	ERSE	-411	It is directly involved in endoplasmic reticulum-associated degradation (ERAD) and targets misfolded glycoproteins for degradation	1,6,16	17,18
EDEM2	NM_018217.2	__	ER	ERSE	- 13 511	Initiates the endoplasmic reticulum-associated degradation (ERAD) that targets misfolded glycoproteins for degradation in an N-glycan-dependent manner.	19–21	
ERLEC1	NM_015701.4	CIM; XTP3-B;	ER lumen	ERSE-26	-255	Probable lectin that binds selectively to improperly folded lumenal proteins.	22	
ERP29	NM_006817.3	ERp28; PDIA9; PDI-DB;	ER	ACGT core	- 95	Plays an important role in the processing of secretory proteins within the endoplasmic reticulum (ER), possibly by participating in the folding of proteins in the ER	23	
ERP44	NM_015051.2	PDIA10; TXNDC4	ER	UPRE<br>ACGT core	- 3 906<br>-260	May have a role in the control of oxidative protein folding in the endoplasmic reticulum.	24	
FKBP2	NM_004470.3	PPIase; FKBP-13	ER membrane	ACGT core<br>UPRE	+ 885<br>-18 617	PPIases accelerate the folding of proteins	1	
FOXO4	NM_005938.3	AFX; AFX1; MLLT7	Cytosol; Nucleus	ERSE-26	- 4662	Transcription factor involved in the regulation of the insulin signaling pathway.	22	
HERPUD1	NM_014685.3	HERP, Mif1, SUP	ER membrane	ERSE II<br>ERSE<br>ACGT core	-167<br>-209<br>-563	Component of the endoplasmic reticulum quality control (ERQC) system involved in ubiquitin-dependent degradation of misfolded endoplasmic reticulum proteins	1,25	25,26
HSP90B1	NM_003299.2	GRP94; GP96; TRA1	ER	ERSE<br>ACGT core	-281<br>-176	Molecular chaperone that functions in the processing and transport of secreted proteins.	5,6,27	17,27
HSPA5	NM_005347.4	BIP; MIF2; GRP78	ER	3x ERSE<br>ERSE-II	-68<br>-107	Involved in the correct folding of proteins and degradation of misfolded proteins	1,6,11,16	16
HYOU1	NM_006389.4	Grp170; HSP12A	ER	UPRE	- 242	Has a pivotal role in cytoprotective cellular mechanisms triggered by oxygen deprivation. May play a role as a molecular chaperone and participate in protein folding.	5,28	18,29
IGF1	NM_001111283.2	MGF; IGFI; IGF-I	Secreted	UPRE<br>ERSE	-4 850<br>+1229	Stimulates glucose transport and is effective at much lower concentrations than insulin, not only regarding glycogen and DNA synthesis but also with regard to enhancing glucose uptake.	30	
LPIN3	NM_001301860.1	SMP2; LIPN3L	ER	ACGT core	+ 92	Regulates fatty acid metabolism.	1,31	
LRFN4	NM_024036.4	SALM3; SALM3.	Plasma Membrane	ERSE-26<br>UPRE	- 7649<br>-6 918	Promotes neurite outgrowth in hippocampal neurons. May play a role in redistributing DLG4 to the cell periphery	22	
MANF	NM_006010.5	ARMET; ARP	ER, Secteted	ERSE-II	+19	Selectively promotes the survival of dopaminergic neurons of the ventral mid-brain. Inhibits cell proliferation and endoplasmic reticulum (ER) stress-induced cell death	6,7	32,33
P4HB	NM_000918.3	PDI; PHDB; PDIA1	ER	ERSE<br>ACGT core	-490	This multifunctional protein catalyzes the formation, breakage and rearrangement of disulfide bonds.	19,34	
PDIA3	NM_005313.4	ERp57, GRP58, ERp60,	ER, secreted (?)	ERSE<br>UPRE	-159<br>-4056	Catalyzes the rearrangement of -S-S- bonds in proteins	11,35	36,37
PDIA4	NM_004911.4	ERP70, ERP72, ERp-72	ER	ERSE<br>UPRE	-12 331<br>+6 829	Catalyzes the rearrangement of -S-S- bonds in proteins	11,38	9
PDIA5	NM_006810.3	PDIR	ER	ACGT core	-131	Catalyzes the rearrangement of -S-S- bonds in proteins. Regulates ER stress-induced ATF6 activation throughdisulfide bond rearrangement, thus blocking ATF6 export from the ER to the Golgi complex, preventing its activation		
PDIA6	NM_001282704.1	PDI-P5; ERP5; TXNDC7	ER lumen\ Plasma membrane	ERSE	-157	May function as a chaperone that inhibits aggregation of misfolded proteins.	5,6,1	40
PPIB	NM_000942.4	OI9; CYPB; CYP-S1	ER	ACGT core	+ 6 782	PPIases accelerate the folding of proteins. It catalyzes the cis-trans isomerization of proline imidic peptide bonds in oligopeptides	11	
RPN1	NM_001179157.1	OST1; RBPH1	ER	ACGT core<br>UPRE	+ 148<br>+521	This protein is involved in the pathway protein glycosylation, which is part of Protein modification.	5,11	
RPN2	NM_001179425.1	SWP1; RPNII;	ER membrane	ACGT core<br>UPRE	+ 4 910<br>-10 171	This gene encodes a type I integral membrane protein found only in the rough endoplasmic reticulum.	5,31	

Name	RefSeq	Alias	Localization	ER response elements	Position realative to the TSS	Function (UniProt)	XBP1 targets (Ref)	ATF6 targets (Ref)
SEC61B	NM_006808.2	--	ER membrane	ACGT core	-795	Necessary for protein translocation in the endoplasmic reticulum. Oligomers of the Sec61 complex form a transmembrane channel where proteins are translocated across and integrated into the ER membrane.	1,31	
SLC33A1	NM_004733.3	AT1; SPG42;	ER; Golgi	ERSE<br>ACGT core	-672<br>+521	Probable acetyl-CoA transporter necessary for O-acetylation of gangliosides. Negatively regulates BMP signaling	1	
SRPRA	NM_003139.3	DP; SRPR; Sralpha	ER membrane	ACGT core<br>UPRE	- 77<br>-6 615	Component of the SRP (signal recognition particle) receptor.	11,41,42	
SRPRB	NM_021203.3	APMCF1	ER membrane	ERSE<br>ACGT core	- 29<br>-33	Component of the SRP (signal recognition particle) receptor.	28	
SSR3	NM_001308197.1	TRAPG	ER membrane	ACGT core<br>UPRE	+ 924	TRAP proteins are part of a complex whose function is to bind calcium to the ER membrane and thereby regulate the retention of ER resident proteins	11,43	
SSR4	NM_001204526.1	CDG1Y; TRAPD	ER membrane	UPRE<br>ACGT core	-6 755<br>-152	TRAP proteins are part of a complex whose function is to bind calcium to the ER membrane and thereby regulate the retention of ER resident proteins	11	
STX5	NM_003164.4	SED5; STX5A	ER, Golgi	ACGT core<br>UPRE	- 12<br>-24	Mediates endoplasmic reticulum to Golgi transport.	1	
SYVN1	NM_032431.2	DER3; HRD1	ER membrane	UPRE<br>ERSE	-557<br>-521	Component of the ER-associated degradation (ERAD) involved in ubiquitin-dependent degradation of misfolded endoplasmic reticulum proteins.	44,45	
VCP	NM_007126.3	p97; TERA; ALS14;	Cytosol/ ER/ Nucleus/ Extracelllular	URPE<br>ACGT core<br>ERSE-26	-452<br>-18 167<br>+1342	Necessary for the fragmentation of Golgi stacks during mitosis and for their reassembly after mitosis. Involved in the formation of the transitional endoplasmic reticulum (tER)	1	
WFS1	NM_001145853.1	WFRS; WFSL; CTRCT41	ER membrane	ERSE-like<br>UPRE	-414<br>-7439	Participates in the regulation of cellular Ca2+ homeostasis, at least partly, by modulating the filling state of the endoplasmic reticulum Ca2+ store	10,37,28	47
XBP1	NM_005080.3	TREB-5, XBP2	ER	ERSE	-78	Functions as a transcription factor during endoplasmic reticulum (ER) stress by regulating the unfolded protein response (UPR).	1,6,48	49,50

References

1 Acosta-Alvear, D. *et al.* XBP1 Controls Diverse Cell Type- and Condition-Specific Transcriptional Regulatory Networks. *Mol. Cell* **27,** 53–66 (2007).

2 Jayanthi, S. *et al.* Methamphetamine induces dopamine D1 receptor-dependent endoplasmic reticulum stress-related molecular events in the rat striatum. *PLoS One* **4,** e6092 (2009).

3 Oda, Y. *et al.* Derlin-2 and Derlin-3 are regulated by the mammalian unfolded protein response and are required for ER-associated degradation. *J. Cell Biol.* **172,** 383–93 (2006).

4 Cross, B. C. S. *et al.* The molecular basis for selective inhibition of unconventional mRNA splicing by an IRE1-binding small molecule. *Proc. Natl. Acad. Sci. U. S. A.* **109,** E869-78 (2012).

5 Wang, Y.-J., Han, D.-Y., Tabib, T., Yates, J. R. & Mu, T.-W. Identification of GABA $_C$ Receptor Protein Homeostasis Network Components from Three Tandem Mass Spectrometry Proteomics Approaches. *J. Proteome Res.* **12,** 5570–5586 (2013).

6 Lee, A.-H., Iwakoshi, N. N. & Glimcher, L. H. XBP-1 regulates a subset of endoplasmic reticulum resident chaperone genes in the unfolded protein response. *Mol. Cell. Biol.* **23,** 7448–59 (2003).

7 Sadighi Akha, A. A. *et al.* Heightened induction of proapoptotic signals in response to endoplasmic reticulum stress in primary fibroblasts from a mouse model of longevity. *J. Biol. Chem.* **286,** 30344–51 (2011).

8 Shen, Y. & Hendershot, L. M. Identification of ERdj3 and OBF-1/BOB-1/OCA-B as direct targets of XBP-1 during plasma cell differentiation. *J. Immunol.* **179,** 2969–78 (2007).

9 Arai, M. *et al.* Transformation-associated gene regulation by ATF6alpha during hepatocarcinogenesis. *FEBS Lett.* **580,** 184–90 (2006).

10 Kanemoto, S. *et al.* XBP1 activates the transcription of its target genes via an ACGT core sequence under ER stress. *Biochem. Biophys. Res. Commun.* **331,** 1146–53 (2005).

11 Shaffer, A. . L. *et al.* XBP1, downstream of Blimp-1, expands the secretory apparatus and other organelles, and increases protein synthesis in plasma cell differentiation. *Immunity* **21,** 81–93 (2004).

12 Muñoz-Lobato, F. *et al.* Protective Role of DNJ-27/ERdj5 in *Caenorhabditis elegans* Models of Human Neurodegenerative Diseases. *Antioxid. Redox Signal.* **20,** 217–235 (2014).

13 Vandewynckel, Y.-P. *et al.* Modulation of the unfolded protein response impedes tumor cell adaptation to proteotoxic stress: a PERK for hepatocellular carcinoma therapy. *Hepatol. Int.* **9,** 93–104 (2015).

14 Zhang, H. M. *et al.* P58 ^{IPK} inhibits coxsackievirus-induced apoptosis via the PI3K/Akt pathway requiring activation of ATF6a and subsequent upregulation of mitofusin 2. *Cell. Microbiol.* **16,** 411–424 (2014).

15 van Huizen, R., Martindale, J. L., Gorospe, M. & Holbrook, N. J. P58IPK, a Novel Endoplasmic Reticulum Stress-inducible Protein and Potential Negative Regulator of eIF2 Signaling. *J. Biol. Chem.* **278,** 15558–15564 (2003).

16 Yoshida, H. *et al.* A time-dependent phase shift in the mammalian unfolded protein response. *Dev. Cell* **4,** 265–71 (2003).

17 Yamamoto, K. *et al.* Transcriptional induction of mammalian ER quality control proteins is mediated by single or combined action of ATF6alpha and XBP1. *Dev. Cell* **13,** 365–76 (2007).

18 Shoulders, M. D. *et al.* Stress-Independent Activation of XBP1s and/or ATF6 Reveals Three Functionally Diverse ER Proteostasis Environments. *Cell Rep.* **3,** 1279–1292 (2013).

19 Wang, S. *et al.* IRE1α-XBP1s induces PDI expression to increase MTP activity for hepatic VLDL assembly and lipid homeostasis. *Cell Metab.* **16,** 473–86 (2012).

20 Olivari, S., Galli, C., Alanen, H., Ruddock, L. & Molinari, M. A Novel Stress-induced EDEM Variant Regulating Endoplasmic Reticulum-associated Glycoprotein Degradation. *J. Biol. Chem.* **280,** 2424–2428 (2005).

21 Badiola, N. *et al.* Induction of ER stress in response to oxygen-glucose deprivation of cortical cultures involves the activation of the PERK and IRE-1 pathways and of caspase-12. *Cell Death Dis.* **2,** e149 (2011).

22 Misiewicz, M. *et al.* Identification of a Novel Endoplasmic Reticulum Stress Response Element Regulated by XBP1. *J. Biol. Chem.* **288,** 20378–20391 (2013).

23 Zhang, B. *et al.* ERp29 is a radiation-responsive gene in IEC-6 cell. *J. Radiat. Res.* **49,** 587–96 (2008).

24 Sha, H. *et al.* Adipocyte spliced form of X-box-binding protein 1 promotes adiponectin multimerization and systemic glucose homeostasis. *Diabetes* **63,** 867–79 (2014).

25 Yamamoto, K., Yoshida, H., Kokame, K., Kaufman, R. J. & Mori, K. Differential contributions of ATF6 and XBP1 to the activation of endoplasmic reticulum stress-responsive cis-acting elements ERSE, UPRE and ERSE-II. *J. Biochem.* **136,** 343–50 (2004).

26 Kokame, K., Kato, H. & Miyata, T. Identification of ERSE-II, a new cis-acting element responsible for the ATF6-dependent mammalian unfolded protein response. *J. Biol. Chem.* **276,** 9199–205 (2001).

27 Marzec, M., Eletto, D. & Argon, Y. GRP94: An HSP90-like protein specialized for protein folding and quality control in the endoplasmic reticulum. *Biochim. Biophys. Acta* **1823,** 774–87 (2012).

28 Kakiuchi, C., Ishiwata, M., Hayashi, A. & Kato, T. XBP1 induces WFS1 through an endoplasmic reticulum stress response element-like motif in SH-SY5Y cells. *J. Neurochem.* **97,** 545–555 (2006).

29 Adachi, Y. *et al.* ATF6 is a transcription factor specializing in the regulation of quality control proteins in the endoplasmic reticulum. *Cell Struct. Funct.* **33,** 75–89 (2008).

30 Hu, M.-C. *et al.* XBP-1, a key regulator of unfolded protein response, activates transcription of IGF1 and Akt phosphorylation in zebrafish embryonic cell line. *Biochem. Biophys. Res. Commun.* **359,** 778–783 (2007).

31 Sriburi, R. *et al.* Coordinate regulation of phospholipid biosynthesis and secretory pathway gene expression in XBP-1(S)-induced endoplasmic reticulum biogenesis. *J. Biol. Chem.* **282,** 7024–34 (2007).

32 Mizobuchi, N. *et al.* ARMET is a soluble ER protein induced by the unfolded protein response via ERSE-II element. *Cell Struct. Funct.* **32,** 41–50 (2007).

33 Oh-Hashi, K., Hirata, Y. & Kiuchi, K. Transcriptional regulation of mouse mesencephalic astrocyte-derived neurotrophic factor in Neuro2a cells. *Cell. Mol. Biol. Lett.* **18,** 398–415 (2013).

34 Rui, Y.-N., Xu, Z., Chen, Z. & Zhang, S. The GST-BHMT assay reveals a distinct mechanism underlying proteasome inhibition-induced macroautophagy in mammalian cells. *Autophagy* **11,** 812–832 (2015).

35 Hetz, C. *et al.* XBP-1 deficiency in the nervous system protects against amyotrophic lateral sclerosis by increasing autophagy. *Genes Dev.* **23,** 2294–306 (2009).

36 Yu, B., Wen, L., Xiao, B., Han, F. & Shi, Y. Single Prolonged Stress induces ATF6 alpha-dependent Endoplasmic reticulum stress and the apoptotic process in medial Frontal Cortex neurons. *BMC Neurosci.* **15,** 115 (2014).

37 Hoffman, S. M. *et al.* Endoplasmic reticulum stress mediates house dust mite-induced airway epithelial apoptosis and fibrosis. *Respir. Res.* **14,** 141 (2013).

38 Savic, S. *et al.* TLR dependent XBP-1 activation induces an autocrine loop in rheumatoid arthritis synoviocytes. *J. Autoimmun.* **50,** 59–66 (2014).

40 Vekich, J. A., Belmont, P. J., Thuerauf, D. J. & Glembotski, C. C. Protein disulfide isomerase-associated 6 is an ATF6-inducible ER stress response protein that protects cardiac myocytes from ischemia/reperfusion-mediated cell death. *J. Mol. Cell. Cardiol.* **53,** 259–267 (2012).

41 Genini, S. *et al.* Strengthening insights into host responses to mastitis infection in ruminants by combining heterogeneous microarray data sources. *BMC Genomics* **12,** 225 (2011).

42 Sriburi, R. *et al.* Coordinate Regulation of Phospholipid Biosynthesis and Secretory Pathway Gene Expression in XBP-1(S)-induced Endoplasmic Reticulum Biogenesis. *J. Biol. Chem.* **282,** 7024–7034 (2006).

43 Odqvist, L. *et al.* NIK controls classical and alternative NF-κB activation and is necessary for the survival of human T-cell lymphoma cells. *Clin. Cancer Res.* **19,** 2319–30 (2013).

44 Kaneko, M. *et al.* A different pathway in the endoplasmic reticulum stress-induced expression of human HRD1 and SEL1 genes. *FEBS Lett.* **581,** 5355–5360 (2007).

45 Yamamoto, K. *et al.* Human HRD1 Promoter Carries a Functional Unfolded Protein Response Element to Which XBP1 but not ATF6 Directly Binds. *J. Biochem.* **144,** 477–486 (2008).

46 Kakiuchi, C., Ishiwata, M., Hayashi, A. & Kato, T. XBP1 induces WFS1 through an endoplasmic reticulum stress response element-like motif in SH-SY5Y cells. *J. Neurochem.* **97,** 545–55 (2006).

47 Odisho, T., Zhang, L. & Volchuk, A. ATF6? regulates the Wfs1 gene and has a cell survival role in the ER stress response in pancreatic ?-cells. *Exp. Cell Res.* **330,** 111–122 (2015).

48 Kakiuchi, C. *et al.* Impaired feedback regulation of XBP1 as a genetic risk factor for bipolar disorder. *Nat. Genet.* **35,** 171–175 (2003).

49 Guo, F.-J. *et al.* ATF6 upregulates XBP1S and inhibits ER stress-mediated apoptosis in osteoarthritis cartilage. *Cell. Signal.* **26,** 332–342 (2014).

50 Yoshida, H. *et al.* ATF6 activated by proteolysis binds in the presence of NF-Y (CBF) directly to the cis-acting element responsible for the mammalian unfolded protein response. *Mol. Cell. Biol.* **20,** 6755–67 (2000).

Table S5. GO enrichment analysis performed on the genes regulated by XBP1, ATF6 and ATF4-NRF2

GO_BP_XBP1

Term	IRE1-mediated unfolded protein response	endoplasmic reticulum unfolded protein response	protein folding	protein N-linked glycosylation	ER-associated ubiquitin-dependent protein catabolic process	ERAD pathway	retrograde protein transport, ER to cytosol	cell redox homeostasis	ER to Golgi vesicle-mediated transport	negative regulation of endoplasmic reticulum stress-induced intrinsic apoptotic signaling pathway	chaperone-mediated protein folding	ATF6-mediated unfolded protein response
Count	53	61	19	9	14	14	9	10	12	6	6	4
%	31,7	36,5	11,4	5,4	8,4	8,4	5,4	6	7,2	3,6	3,6	2,4
p-value	7,50E-96	4,30E-92	1,60E-12	1,10E-06	1,20E-13	1,30E-12	5,50E-11	2,30E-08	4,50E-07	6,70E-07	9,00E-05	7,50E-05
Benj	2,50E-92	7,30E-89	3,30E-10	7,00E-05	2,90E-11	2,90E-10	9,40E-09	2,30E-06	3,30E-05	4,70E-05	3,40E-03	2,90E-03
Genes	ARFGAP1	ARFGAP1	ALG12	EDEM1	DNAJB9	DNAJB9	EDEM1	DNAJC10	ARFGAP1	XBP1	FKBP14	XBP1
	ATP6V0D1	ATP6V0D1	DNAJB11	EDEM2	DNAJC10	DNAJC10	EDEM2	ERP44	SEC31A	HSPA1A	FKBP2	HSP90B1
	CTDSP2	CCL2	DNAJC10	STT3A	EDEM1	EDEM1	SEC61B	IL6	YIF1A	HERPUD1	CANX	HSPA5
	CXXC1	CXCL8	FKBP14	DAD1	EDEM2	EDEM2	DERL1	NOS2	CREB3L2	HYOU1	CRTAP	WFS1
	DDX11	CTDSP2	FKBP2	RPN1	SEC61B	SEC61B	ERLEC1	P4HB	COPG1	SYVN1	PPIB	
	DNAJB11	CXXC1	CANX	RPN2	XBP1	XBP1	HSP90B1	PDIA3	DCTN1	WFS1	PDIA4	
	DNAJB9	DDX11	CRTAP	SYVN1	DERL1	DERL1	HERPUD1	PDIA4	GOSR2			
	DNAJC3	DNAJB11	ERP29	VCP	ERLEC1	ERLEC1	SYVN1	PDIA5	HYOU1			
	EDEM1	DNAJB9	ERP44		HSP90B1	HSP90B1	VCP	PDIA6	PREB			
	FKBP14	DNAJC3	HSP90B1		HSPA5	HSPA5		TXNDC11	SPTBN5			
	KDELR3	EDEM1	HSPA1A		HERPUD1	HERPUD1			STX5			
	SEC31A	EDEM2	HSPA5		SYVN1	SYVN1			VCP			
	SEC63	FKBP14	PPIB		VCP	VCP						
	SHC1	KDELR3	PDIA3		WFS1	WFS1						
	SRPRA	SEC31A	PDIA4									
	SRPRB	SEC63	PDIA5									
	SEC61A1	SHC1	PDIA6									
	SEC61B	SRPRA	TXNDC11									
	TSPYL2	SRPRB	WFS1									
	TATDN2	SEC61A1										
	WIPI1	SEC61B										
	XBP1	TSPYL2										
	YIF1A	TATDN2										
	ACADVL	WIPI1										
	ADD1	XBP1										
	BFAR	YIF1A										
	CUL7	ACADVL										
	DCTN1	ADD1										
	ERN1	BFAR										
	EXTL3	CREB3L2										
	GFPT1	CUL7										
	GSK3A	DERL1										
	GOSR2	DCTN1										
	HSPA1A	ERN1										
	HSPA5	EXTL3										
	HDGF	GFPT1										
	HYOU1	GSK3A										
	KLHDC3	GOSR2										
	LMNA	HSP90B1										
	MYDGF	HSPA1A										
	PLA2G4B	HSPA5										
	PREB	HDGF										
	PDIA5	HERPUD1										
	PDIA6	HYOU1										
	PPP2R5B	KLHDC3										
	SSR1	LMNA										
	SERP1	MYDGF										
	SULT1A3	PLA2G4B										
	SYVN1	PREB										
	TLN1	PDIA5										
	TPP1	PDIA6										
	WFS1	PPP2R5B										
	ZBTB17	SSR1										
		SERP1										
		SULT1A3										
		SYVN1										
		TLN1										
		TPP1										
		VCP										
		WFS1										
		ZBTB17										

GO_CC_XBP1

Term	endoplasmic reticulum	endoplasmic reticulum membrane	endoplasmic reticulum lumen	endoplasmic reticulum chaperone complex	cytoplasm		rough endoplasmic reticulum	endoplasmic reticulum-Golgi intermediate compartment	smooth endoplasmic reticulum	endoplasmic reticulum quality control compartment	oligosaccharyltransferase complex
Count	65	47	21	8	130		10	10	7	4	4
%	38,9	28,1	12,6	4,8	77,8		6	6	4,2	2,4	2,4
p-value	8,10E-27	8,10E-22	6,40E-16	9,40E-13	2,00E-10		9,30E-09	4,70E-07	3,30E-07	1,70E-04	7,10E-05
Benj	1,70E-24	8,40E-20	4,60E-14	4,90E-11	7,40E-09		2,90E-07	8,80E-06	7,10E-06	2,00E-03	1,00E-03
Genes	ALG12	ALG12	DNAJB11	DNAJB11	ARFGAP1	IGF1	CCL2	YIF1A	DNAJC3	EDEM1	STT3A
	ALG2	ALG2	DNAJB9	DNAJC10	ALG12	IL6	DNAJC3	ERP44	CANX	EDEM2	DAD1
	CCL2	DNAJB9	DNAJC10	HSP90B1	ALG2	KLHDC3	SRPRA	GJB2	ERP29	ERLEC1	RPN1
	DNAJB11	DNAJC3	DNAJC3	HSPA5	ATP6V0D1	LMNA	SRPRB	GOSR2	HSPA5	SYVN1	RPN2
	DNAJB9	EDEM1	EDEM2	HYOU1	CCL2	LPIN3	SEC61A1	HSPA5	HYOU1		
	DNAJC10	FKBP14	FKBP14	PPIB	CXXC1	MANF	SEC61B	MYDGF	PPIB		
	DNAJC3	FKBP2	CANX	P4HB	DDX11	MYDGF	CANX	NUCB2	SYVN1		
	EDEM1	KDELR3	CRTAP	PDIA6	DNAJB11	NOS2	RPN1	P4HB			
	EDEM2	SEC31A	ERLEC1		DNAJB9	Nucb2	RPN2	PDIA6			
	FKBP14	SEC63	ERP29		DNAJC10	NUDT9	SSR4	STX5			
	FKBP2	SRPRA	ERP44		DNAJC3	PPIB					
	KDELR3	SRPRB	HSP90B1		EDEM1	PECR					
	SEC31A	STT3A	HSPA5		EDEM2	PLA2G4B					
	SEC63	SEC61A1	HYOU1		FKBP14	PRNP					
	SRPRA	SEC61B	MYDGF		FKBP2	PREB					
	SRPRB	XBP1	PPIB		GNAQ	P4HB					
	STT3A	YIF1A	P4HB		KDELR3	PRM3					
	SEC61A1	BFAR	PDIA3		NAT6	PDIA3					
	SEC61B	CREB3L2	PDIA4		NLRP4	PDIA4					
	XBP1	CANX	PDIA5		SEC31A	PDIA5					
	YIF1A	COPG1	PDIA6		SEC63	PDIA6					
	BFAR	DAD1			SERBP1	PPP1R3G					
	CREB3L2	DERL1			SHC1	PPP2R5B					
	CANX	ERP44			SPG7	REEP5					
	CRTAP	ERN1			SRPRA	RPN1					
	COPG1	EXTL3			SRPRB	RPN2					
	DAD1	GOSR2			STT3A	SESN2					
	DERL1	HSP90B1			SEC61A1	SSR1					
	ERLEC1	HSPA5			SEC61B	SSR3					
	ERP29	HERPUD1			TSPYL2	SSR4					
	ERP44	LPIN3			TATDN2	SLC33A1					
	ERN1	PREB			WIPI1	SPTBN5					
	EXTL3	PDIA5			XBP1	SMPD1					
	GOSR2	PDIA6			YIF1A	SERP1					
	HSP90B1	RPN1			ACADVL	SULT1A3					
	HSPA13	RPN2			ADD1	SOD2					
	HSPA5	SSR1			ALOX12B	SYVN1					
	HERPUD1	SSR3			ATG13	STX5					
	HYOU1	SSR4			BFAR	TLN1					
	LPIN3	SLC33A1			CREB3L2	TXNDC11					
	MANF	SERP1			CANX	TIMM44					
	MYDGF	SYVN1			CHST7	TMCO1					
	NUCB2	STX5			CRTAP	TMEM168					
	PPIB	TXNDC11			CAV2	TMEM175					
	PRNP	TMCO1			CHKB	TGS1					
	PREB	VCP			COPG1	TPP1					
	P4HB	WFS1			CKMT2	USP34					
	PDIA3				CUL7	USP4					
	PDIA4				DAD1	VCP					
	PDIA5				DERL1	VEGFA					
	PDIA6				DCTN1	WFS1					
	REEP5				EML1	ZHX2					
	RPN1				ERLEC1	ZPBP2					
	RPN2				ERP29						
	SSR1				ERP44						
	SSR3				ERN1						
	SSR4				ESR1						
	SLC33A1				EIF2A						
	SERP1				EXTL3						
	SYVN1				FOXO1						
	STX5				FOXO4						
	TXNDC11				GJB2						
	TMCO1				GAD1						
	VCP				GAD2						
	WFS1				GFPT1						
					GSK3A						
					GOSR2						
					GADD45B						
					HSP90B1						
					HSPA13						
					HSPA1A						
					HSPA5						
					HDGF						
					HERPUD1						
					HYAL3						
					HADH						
					HYOU1						

GO_MF_XBP1

Term	intramolecular oxidoreductase activity	misfolded protein binding	isomerase activity	unfolded protein binding	chaperone binding	oligosaccharyl transferase activity
Count	8	5	11	9	8	4
%	4,8	3	6,6	5,4	4,8	2,4
p-value	4,50E-07	2,70E-06	2,10E-06	5,80E-06	7,30E-06	7,70E-05
Benj	1,40E-04	4,10E-04	4,20E-04	7,10E-04	7,40E-04	5,80E-03
Genes	ERP29	DNAJB9	FKBP14	DNAJB11	DNAJC10	STT3A
	ERP44	DNAJC10	FKBP2	SPG7	DNAJC3	DAD1
	P4HB	DNAJC3	ERP29	CANX	ERP29	RPN1
	PDIA3	EDEM1	ERP44	ERLEC1	HSPA5	RPN2
	PDIA4	HSPA5	PPIB	HSP90B1	HYOU1	
	PDIA5		P4HB	HSPA1A	PRNP	
	PDIA6		PDIA3	HSPA5	SYVN1	
	TXNDC11		PDIA4	PPIB	TIMM44	
			PDIA5	SYVN1		
			PDIA6			
			TXNDC11			

GO_BP_ATF6

Term	endoplasmic reticulum unfolded protein response	ATF6-mediated unfolded protein response	ER-associated ubiquitin-dependent protein catabolic process	IRE1-mediated unfolded protein response	protein folding	regulation of sequence-specific DNA binding transcription factor activity	negative regulation of endoplasmic reticulum stress-induced intrinsic apoptotic signaling pathway	regulation of retrograde protein transport, ER to cytosol
Count	17	8	11	9	9	12	5	3
%	27,9	13,1	18	14,8	14,8	19,7	8,2	4,9
p-value	5,00E-21	4,80E-16	9,80E-14	9,80E-11	1,10E-06	5,00E-08	6,00E-07	8,90E-04
Benj	1,90E-18	9,00E-14	1,70E-11	8,40E-09	3,20E-05	2,80E-06	1,90E-05	1,00E-02
Genes	DNAJB11	XBP1	EDEM1	DNAJB11	DNAJB11	CRTC2	XBP1	EDEM1
	DNAJC3	ATF6B	FBXO6	DNAJC3	CALR	CREBZF	HERPUD1	OS9
	EDEM1	CALR	OS9	EDEM1	HSP90B1	TAF1	HYOU1	DERL3
	WIPI1	HSP90B1	SEL1L	WIPI1	HSPA5	MBTPS2	PDX1	
	XBP1	HSPA5	XBP1	XBP1	NFYC	MAPK1	WFS1	
	ATF6B	MBTPS1	DERL3	HSPA5	PDIA3	MAPK11		
	CREB3L3	MBTPS2	HSP90B1	HYOU1	PDIA4	MAPK14		
	CALR	WFS1	HSPA5	PDIA6	PDIA6	MAPK3		
	DERL3		HERPUD1	WFS1	WFS1	NR0B2		
	HSP90B1		SDF2L1			SRF		
	HSPA5		WFS1			UBC		
	HERPUD1					WFS1		
	HYOU1							
	MBTPS1							
	MBTPS2							
	PDIA6							
	WFS1							

GO_CC_ATF6

Term	endoplasmic reticulum	endoplasmic reticulum lumen	endoplasmic reticulum membrane	nuclear outer membrane-endoplasmic reticulum membrane network	cytoplasm	smooth endoplasmic reticulum	Golgi apparatus	nuclear transcription factor complex	endocytic vesicle lumen	nucleus
Count	31	13	20	20	54	5	16	5	3	34
%	50,8	21,3	32,8	32,8	88,5	8,2	26,2	8,2	4,9	55,7
p-value	3,40E-16	2,20E-12	2,20E-10	3,20E-10	7,00E-07	4,00E-06	6,70E-05	9,40E-04	1,40E-03	7,40E-03
Benj	9,80E-14	2,10E-10	9,30E-09	1,20E-08	1,10E-05	5,40E-05	7,60E-04	8,30E-03	1,10E-02	4,00E-02
Genes	ATP2A2	DNAJB11	ATP2A2	ATP2A2	ATP2A2	DNAJC3	TNFRSF1A	CEBPB	CALR	CEBPB
	DNAJB11	DNAJC3	DNAJC3	DNAJC3	CEBPB	APP	WIPI1	TAF1	HSP90B1	CRTC2
	DNAJC3	OS9	EDEM1	EDEM1	CRTC2	CALR	ATF6B	CREB1	HYOU1	CREBZF
	EDEM1	BGLAP	ORMDL2	ORMDL2	DNAJB11	HSPA5	APP	NFYA		DNAJB11
	FBXO6	CALR	OS9	OS9	DNAJC3	HYOU1	BGLAP	NFYC		MAFA
	ORMDL2	HSP90B1	SEL1L	SEL1L	EDEM1		CALR			MAX
	OS9	HSPA5	XBP1	XBP1	FBXO6		CRELD2			TAF1
	SEL1L	HYOU1	ATF6B	ATF6B	MAX		MBTPS1			XBP1
	XBP1	MBTPS1	CREB3L3	CREB3L3	ORMDL2		MBTPS2			YY1
	ATF6B	PDIA3	CALR	CALR	OS9		MAPK1			ATF6B
	APP	PDIA4	DERL3	DERL3	SEL1L		MAPK3			APP
	BGLAP	PDIA6	HSP90B1	HSP90B1	TNFRSF1A		NUCB1			CREB1
	CREB3L3	SDF2L1	HSPA5	HSPA5	WIPI1		GALNT3			CREB3L3
	CALR		HERPUD1	HERPUD1	XBP1		RTN3			CALR
	CRELD2		MBTPS1	MBTPS1	YY1		SAR1A			GTF2I
	DERL3		PDIA6	PDIA6	ATF6B		SREBF2			HSP90B1
	HSP90B1		RTN3	RTN3	APP					HSPA5
	HSPA5		SREBF2	SREBF2	BPGM					HNF4A
	HERPUD1		SDF2L1	SDF2L1	BGLAP					MANF
	HYOU1		WFS1	WFS1	CREB1					MAPK1
	MBTPS1				CREB3L3					MAPK11
	MANF				CALR					MAPK14
	NUCB1				CRELD2					MAPK3
	PDIA3				DERL3					NR0B2
	PDIA4				DDC					NFYA
	PDIA6				GTF2I					NFYC
	RTN3				HSP90B1					NUCB1
	SAR1A				HSPA5					PDX1
	SREBF2				HNF4A					PDIA3
	SDF2L1				HERPUD1					SRF
	WFS1				HYOU1					SUMO2
					MBTPS1					SREBF2
					MBTPS2					UBC
					MANF					MYC
					MAPK1					
					MAPK11					
					MAPK14					
					MAPK3					
					NNMT					
					NR0B2					
					NUCB1					
					PDX1					
					GALNT3					
					PDIA3					
					PDIA4					
					PDIA6					
					RTN3					
					SAR1A					
					SRF					
					SREBF2					
					SDF2L1					
					UBC					
					MYC					
					WFS1					

GO_MF_ATF6

Term	transcription factor binding	transcription factor activity, sequence-specific DNA binding	enzyme binding	protein binding	misfolded protein binding	chaperone binding	glycoprotein binding
Count	14	18	20	54	4	5	5
%	23	29,5	32,8	88,5	6,6	8,2	8,2
p-value	0,000000018	0,00000061	0,0000059	0,0000063	0,0000087	0,00018	0,00046
Benj	0,0000059	0,00002	0,00014	0,00014	0,00018	0,0023	0,0049
Genes	CEBPB	CEBPB	ATP2A2	ATP2A2	DNAJC3	DNAJC3	EDEM1
	CRTC2	CREBZF	CEBPB	CEBPB	EDEM1	CALR	FBXO6
	TAF1	MAFA	DNAJC3	CRTC2	HSPA5	HSPA5	OS9
	XBP1	MAX	OS9	CREBZF	SDF2L1	HYOU1	CALR
	CREB1	TAF1	XBP1	DNAJB11		SDF2L1	HSPA5
	HNF4A	XBP1	APP	DNAJC3			
	MAPK1	YY1	CREB1	EDEM1			
	MAPK14	ATF6B	CALR	FBXO6			
	NR0B2	CREB1	DDC	MAFA			
	NFYC	CREB3L3	GTF2I	MAX			
	PDX1	GTF2I	HSP90B1	OS9			
	SRF	HNF4A	HSPA5	SEL1L			
	MYC	NFYA	MAPK1	TAF1			
	WFS1	NFYC	MAPK14	TNFRSF1A			
		PDX1	MAPK3	WIPI1			
		SRF	SRF	WBP1			
		SREBF2	SUMO2	XBP1			
		MYC	SDF2L1	YY1			
			UBC	ATF6B			
			WFS1	APP			
				CREB1			
				CREB3L3			
				CALR			
				CRELD2			
				DERL3			
				DDC			
				GTF2I			
				HSP90B1			
				HSPA5			
				HNF4A			
				HERPUD1			
				HYOU1			
				MANF			
				MAPK1			
				MAPK11			
				MAPK14			
				MAPK3			
				NR0B2			
				NFYA			
				NFYC			
				NUCB1			
				PDX1			
				PDIA3			
				PDIA4			
				PDIA6			
				RTN3			
				SAR1A			
				SRF			
				SUMO2			
				SREBF2			
				SDF2L1			
				UBC			
				MYC			
				WFS1			

GO_BP_ATF4

Term	PERK-mediated unfolded protein response	endoplasmic reticulum unfolded protein response	response to amino acid	cellular response to hypoxia	intrinsic apoptotic signaling pathway in response to endoplasmic reticulum stress	cellular response to glucose starvation	negative regulation of translational initiation in response to stress
Count	12	17	13	14	8	6	3
%	8	11,3	8,7	9,3	5,3	4	2
p-value	5,10E-18	3,40E-14	4,30E-11	1,40E-10	0,00000071	0,0000048	0,0016
Benj	9,00E-16	2,60E-12	1,80E-09	0,000000005	0,000012	0,000066	0,011
Genes	CCL2	CCL2	CCL2	BNIP3L	CEBPB	ATF4	ATF4
	CXCL8	CXCL8	CEBPB	CREBBP	CHAC1	ASNS	EIF2AK3
	DDIT3	DDIT3	ASNS	CA9	DDIT3	EIF2AK3	EIF2S1
	ATF3	ATF3	EGFR	EGR1	TNFRSF10B	HSPA5	
	ATF4	ATF4	FGF21	EIF4EBP1	ATF4	NFE2L2	
	ASNS	ATF6	GCLC	HMOX1	HERPUD1	TP53	
	EIF2AK3	ASNS	GCLM	HIF1A	SIRT1		
	EIF2S1	CTH	GSTP1	MTOR	TRIB3		
	HSPA5	EIF2AK2	GSS	NFE2L2			
	HERPUD1	EIF2AK3	IL6	PTEN			
	IGFBP1	EIF2S1	MMP2	SIRT1			
	NFE2L2	FGF21	MTOR	SIRT2			
		HSPA5	MTHFR	TP53			
		HERPUD1		VEGFA			
		IGFBP1					
		NFE2L2					
		PARP16					

GO_CC_ATF4

Term	cytoplasm		extracellular region	mitochondrion	endoplasmic reticulum	extracellular matrix	plasma membrane region	nucleus	nuclear transcription factor complex	endoplasmic reticulum membrane	nuclear outer membrane-endoplasmic reticulum membrane network
Count	120		70	34	29	14	19	77	7	18	18
%	80		46,7	22,7	19,3	9,3	12,7	51,3	4,7	12	12
p-value	2,50E-09		5,80E-09	0,0000011	0,00011	0,00032	0,00046	0,00052	0,00066	0,0025	0,0031
Benj	0,00000031		0,00000048	0,000045	0,0025	0,0055	0,0066	0,0071	0,0086	0,022	0,026
Genes	MCL1	MTHFD2	BTG2	MCL1	BNIP3L	WNT10A	S100P	MCL1	CEBPB	UGT2B7	UGT2B7
	BNIP3L	MTHFR	CCL2	BNIP3L	CCL2	APOE	ATF4	BNIP3L	DDIT3	ATF6	ATF6
	BRCA1	MGST1	CXCL8	DDIT4	UGT2B7	DST	CLSTN2	BRCA1	RB1	CLSTN2	CLSTN2
	BTG2	MAP1LC3A	FAS	NARS	ATF6	HSPA5	CA9	CEBPB	ATF4	CAV1	CAV1
	CCL2	MAP1LC3B	NQO1	CASP8	APOE	LTBP1	CAV1	CREBBP	HIF1A	CFTR	CFTR
	CEBPB	MAPK1	RAB7A	CAT	BGLAP	LOXL1	CFTR	DDIT3	STAT3	DST	DST
	CREBBP	MAPK3	S100P	COX6A2	CLSTN2	MMP2	DDR2	FAS	TP53	EGFR	EGFR
	CHAC1	NOS3	UGT2B7	DISC1	CAT	PRDX1	DISC1	NDC80		EIF2AK3	EIF2AK3
	DDIT3	NFKB1	WNT10A	GSTP1	CAV1	SERPINF1	DST	RB1		HSPA5	HSPA5
	DDIT4	NFE2L2	APOE	GSR	CFTR	SLC25A5	EGFR	POLR2C		HMOX1	HMOX1
	FAS	NUPR1	NARS	HSPA5	DISC1	SPON2	HMOX1	S100P		HERPUD1	HERPUD1
	NQO1	OBSCN	BGLAP	IDH1	DST	SOD1	IL6R	SOX21		LCLAT1	LCLAT1
	NDC80	OVGP1	CAT	ME1	ERP29	TGFBI	MAPK1	ATF3		MTOR	MTOR
	RAB7A	PRDX1	CTH	MMP2	EGFR	VEGFA	MAPK3	ATF4		MGST1	MGST1
	POLR2C	PTEN	CFTR	MTOR	EIF2AK3		NOS3	ATF5		PARP16	PARP16
	S100P	PIK3CA	DDR2	MTHFD2	FGFR3		PTEN	ATF6		PKD2	PKD2
	UGT2B7	PGD	DST	MGST1	HSPA5		PKD2	APOE		RHOA	RHOA
	ATF4	PSAT1	ERP29	MAP1LC3B	HMOX1		SIGMAR1	ATG16L2		SIGMAR1	SIGMAR1
	ATF5	PARP16	EGFR	MAPK1	HERPUD1		SLC3A2	BIRC2			
	ATF6	PKD2	EIF2S1	MAPK3	KEAP1			CA9			
	APOE	RHOA	EIF2A	NFKB1	LCLAT1			CASP8			
	ASNS	RCN1	FTL	PRDX1	MTOR			CTH			
	NARS	SHMT2	FGF19	PTEN	MGST1			DISC1			
	ATG16L2	SERPINF1	FGF2	SHMT2	PARP16			DST			
	BIRC2	SIGMAR1	FGF21	STAT3	PKD2			EGR1			
	BGLAP	STAT3	FGFR3	SIRT1	RHOA			EGFR			
	CLSTN2	SIRT1	G6PD	SLC25A36	RCN1			ETNK1			
	CASP8	SIRT2	GSTA1	SLC25A5	SIGMAR1			EIF1			
	CAT	SLC1A4	GSTA3	SDHB	TP53			EIF2AK2			
	CAV1	SLC25A36	GSTA5	SOD1				EIF2S1			
	CTH	SLC25A5	GSTM3	SOD2				EIF4EBP1			
	CARS	SLC3A2	GSTP1	TXNRD1				FGF2			
	CFTR	SDHB	GPX2	TP53				FGFR3			
	COX6A2	SOD1	GSS	UCP1				FOXD3			
	DISC1	SOD2	GSR					FOXP2			
	DST	TXNRD1	HSPA5					G6PD			
	EGR1	TGFBI	HMOX1					GSTM3			
	ERP29	TRIB1	HBB					GSTP1			
	EGFR	TRIB3	IGFBP1					HSPA5			
	ETNK1	TP53	IL6R					HHEX			
	EIF1	UCP1	IL6					HMOX1			
	EIF2AK2	VEGFA	IDH1					HES5			
	EIF2AK3		KRT16					HOXB7			
	EIF2S1		LTBP1					HOXD11			
	EIF2A		LOXL1					HIF1A			
	EIF4EBP1		MMP2					IRF7			
	FTL		MTHFD2					KEAP1			
	FGF2		MAPK1					KRT16			
	FGFR3		MAPK3					KMT2D			
	G6PD		OVGP1					MMP2			
	GCLC		PRDX1					MTOR			
	GCLM		PTEN					MED29			
	GSTA1		PGD					MGST1			
	GSTA3		PSAT1					MAPK1			
	GSTA5		PLAU					MAPK3			
	GSTM1		PKD2					NOS3			
	GSTM3		RHOA					NFKB1			
	GSTP1		SHMT2					NFE2L2			
	GPX2		SERPINF1					NUPR1			
	GSS		SERPINI1					PRDX1			
	GSR		SLC1A4					PHACTR3			
	HSPA5		SLC25A5					PTEN			
	HHEX		SLC3A2					PGD			
	HMOX1		SPON2					PARP16			
	HBB		SDHB					SHMT2			
	HOXB7		SOD1					SIGMAR1			
	HERPUD1		SOD2					STAT3			
	HIF1A		TXNRD1					SIRT1			
	IRF7		TGFBI					SIRT2			
	IL6		VEGFA					SLC25A5			
	IDH1							SLC3A2			
	KEAP1							SDHB			
	LCLAT1							SOD1			
	LOXL1							TXNRD1			
	ME1							TRIB1			
	MGAT3							TRIB3			
	MMP2							TP53			
	MTOR										

GO_MF_ATF4

Term	identical protein binding	antioxidant activity	protein dimerization activity	enzyme binding	ubiquitin-like protein ligase binding	transcription factor binding	catalytic activity	protein binding		receptor binding	phosphatase binding	macromolecular complex binding	kinase activity
Count	44	13	37	40	14	18	80	117		29	9	26	18
%	29,3	8,7	24,7	26,7	9,3	12	53,3	78		19,3	6	17,3	12
p-value	2,60E-14	4,70E-12	1,00E-11	0,000000034	0,0000015	0,0000021	0,0000037	0,000036		0,000049	0,000058	0,00013	0,0026
Benj	1,90E-11	1,70E-09	2,50E-09	0,0000051	0,00011	0,00012	0,00016	0,0012		0,0013	0,0014	0,003	0,032
Genes	MCL1	NQO1	MCL1	BRCA1	BRCA1	CEBPB	BRCA1	MCL1	MTHFR	BRCA1	EGFR	CEBPB	CCL2
	BNIP3L	APOE	BNIP3L	CEBPB	CEBPB	CREBBP	CCL2	BNIP3L	MGST1	CCL2	EIF2AK3	CREBBP	DDR2
	CEBPB	CAT	CEBPB	FAS	RB1	DDIT3	CREBBP	BRCA1	MAP1LC3A	CXCL8	EIF4EBP1	ATF5	EGFR
	DDIT3	GSTA1	DDIT3	RAB7A	ATF6	RB1	CHAC1	BTG2	MAP1LC3B	CEBPB	MAPK1	APOE	ETNK1
	FAS	GSTP1	POLR2C	RB1	CASP8	ATF4	NQO1	CCL2	MAPK1	CHAC1	MAPK3	CASP8	EIF2AK2
	NQO1	GPX2	ATF3	ATF5	EGFR	HHEX	RAB7A	CXCL8	MAPK3	RB1	PHACTR3	DDR2	EIF2AK3
	NDC80	GSR	ATF4	ATF6	HSPA5	HES5	POLR2C	CEBPB	NOS3	S100P	STAT3	DISC1	FGF19
	RB1	HBB	ATF6	CASP8	HIF1A	HIF1A	UGT2B7	CREBBP	NFKB1	WNT10A	SOD1	DST	FGF2
	ATF3	MGST1	APOE	CAT	MAP1LC3A	KEAP1	ASNS	CHAC1	NFE2L2	APOE	TP53	EGR1	FGFR3
	ATF6	PRDX1	ASNS	CAV1	MAP1LC3B	MTOR	NARS	DDIT3	OBSCN	CASP8		EGFR	LTBP1
	APOE	SOD1	CAT	CFTR	SLC25A5	MAPK1	BIRC2	DDIT4	PRDX1	CAT		EIF2S1	MTOR
	ASNS	SOD2	CAV1	EGR1	TRIB1	NFKB1	CA9	FAS	PHACTR3	CAV1		EIF2A	MAPK1
	CASP8	TXNRD1	CARS	EGFR	TRIB3	NFE2L2	CASP8	NQO1	PTEN	DST		HSPA5	MAPK3
	CAT		DST	EIF2AK3	TP53	STAT3	CAT	NDC80	PIK3CA	EGFR		HHEX	OBSCN
	CAV1		ERP29	EIF4EBP1		SIRT1	CTH	RAB7A	PLAU	FGF19		HES5	PIK3CA
	CTH		EGFR	GCLM		SIRT2	CARS	RB1	PARP16	FGF2		MTOR	PLAU
	CARS		EIF2AK3	GSTM1		TRIB1	CFTR	POLR2C	PKD2	FGF21		MTHFR	TRIB1
	DST		FOXP2	GSTM3		TP53	COX6A2	S100P	RHOA	HIF1A		NFKB1	TRIB3
	ERP29		G6PD	GSTP1			DDR2	TNFRSF10B	RCN1	IGFBP1		NUPR1	
	EGFR		GCLC	HSPA5			DST	WNT10A	SHMT2	IL6R		PTEN	
	EIF2AK3		GCLM	HMOX1			ERP29	ATF3	SERPINF1	IL6		SHMT2	
	FTL		GSTM1	HIF1A			EGFR	ATF4	STAT3	IDH1		STAT3	
	FOXP2		GSTM3	IL6R			ETNK1	ATF5	SIRT1	PTEN		SIRT1	
	G6PD		GSS	MTOR			EIF2AK2	ATF6	SIRT2	PKD2		SIRT2	
	GSTM1		HHEX	MAP1LC3A			EIF2AK3	APOE	SLC25A5	STAT3		TGFBI	
	GSTM3		HMOX1	MAP1LC3B			FGF19	ASNS	SLC3A2	SIRT1		TP53	
	GSS		HES5	MAPK1			FGF2	BIRC2	SPON2	TGFBI			
	HHEX		HIF1A	MAPK3			FGFR3	CASP8	SDHB	TP53			
	HMOX1		IL6R	PHACTR3			G6PD	CAT	SOD1	VEGFA			
	IL6R		IDH1	PTEN			GCLC	CAV1	SOD2				
	IDH1		MGST1	PARP16			GCLM	CCDC130	TXNRD1				
	MGST1		NFKB1	PKD2			GSTA1	CTH	TGFBI				
	MAPK1		PKD2	STAT3			GSTA3	CARS	TRIB1				
	NFKB1		STAT3	SIRT1			GSTA5	CFTR	TRIB3				
	PRDX1		SOD1	SIRT2			GSTM1	DDR2	TP53				
	PTEN		TP53	SLC25A5			GSTM3	DISC1	VEGFA				
	PKD2		VEGFA	SOD1			GSTP1	DST					
	SHMT2			TRIB1			GPX2	EGR1					
	STAT3			TRIB3			GSS	ERP29					
	SIRT1			TP53			GSR	EGFR					
	SOD1						HSPA5	ETNK1					
	SOD2						HMOX1	EIF2AK2					
	TP53						HBB	EIF2AK3					
	VEGFA						IDH1	EIF2S1					
							KEAP1	EIF2A					
							LTBP1	EIF4EBP1					
							KMT2D	FTL					
							LCLAT1	FGF19					
							LOXL1	FGF2					
							ME1	FGF21					
							MGAT3	FGFR3					
							MMP2	FOXD3					
							MTOR	FOXP2					
							MTHFD2	G6PD					
							MTHFR	GCLC					
							MGST1	GCLM					
							MAPK1	GSTM1					
							MAPK3	GSTM3					
							NOS3	GSTP1					
							OBSCN	GSS					
							OVGP1	HSPA5					
							PRDX1	HHEX					
							PTEN	HMOX1					
							PIK3CA	HBB					
							PGD	HES5					
							PSAT1	HOXB7					
							PLAU	HERPUD1					
							PARP16	HIF1A					
							RHOA	IGFBP1					
							SHMT2	IRF7					
							SIRT1	IL6R					
							SIRT2	IL6					
							SLC3A2	IDH1					
							SDHB	KEAP1					
							SOD1	KRT16					
							SOD2	LTBP1					
							TXNRD1	KMT2D					
							TRIB1	LCLAT1					
							TRIB3	MMP2					
							ZC3H12C	MTOR					
								MED29					
